# Multilayered regulation of secondary metabolism in medicinal plants

**DOI:** 10.1186/s43897-023-00059-y

**Published:** 2023-06-06

**Authors:** Yan Zhao, Guanze Liu, Feng Yang, Yanli Liang, Qingqing Gao, Chunfan Xiang, Xia Li, Run Yang, Guanghui Zhang, Huifeng Jiang, Lei Yu, Shengchao Yang

**Affiliations:** 1 Key Laboratory of Medicinal Plant Biology of Yunnan Province, National & Local Joint Engineering Research Center on Germplasms Innovation & Utilization of Chinese Medicinal Materials in Southwest China, Yunnan Agricultural University, 650201 Kunming, China; 2 College of Agronomy & Biotechnology, Yunnan Agricultural University, Kunming, 650201 China; 3 Institute of Chinese Medicinal Materials, Nanjing Agricultural University, Nanjing, 210095 China; 4 Key Laboratory of Engineering Biology for Low-Carbon Manufacturing, Tianjin Institute of Industrial Biotechnology, Chinese Academy of Sciences, Tianjin, 300308 China; 5 College of Agronomy, Yunnan Urban Agricultural Engineering and Technological Research Center, Kunming University, Kunming, 650214 China

**Keywords:** Medicinal plants, Secondary metabolism, Multilayered regulation, Transcription factors, Epigenetic regulation

## Abstract

Medicinal plants represent a huge reservoir of secondary metabolites (SMs), substances with significant pharmaceutical and industrial potential. However, obtaining secondary metabolites remains a challenge due to their low-yield accumulation in medicinal plants; moreover, these secondary metabolites are produced through tightly coordinated pathways involving many spatiotemporally and environmentally regulated steps. The first regulatory layer involves a complex network of transcription factors; a second, more recently discovered layer of complexity in the regulation of SMs is epigenetic modification, such as DNA methylation, histone modification and small RNA-based mechanisms, which can jointly or separately influence secondary metabolites by regulating gene expression. Here, we summarize the findings in the fields of genetic and epigenetic regulation with a special emphasis on SMs in medicinal plants, providing a new perspective on the multiple layers of regulation of gene expression.

**Graphical Abstract:**

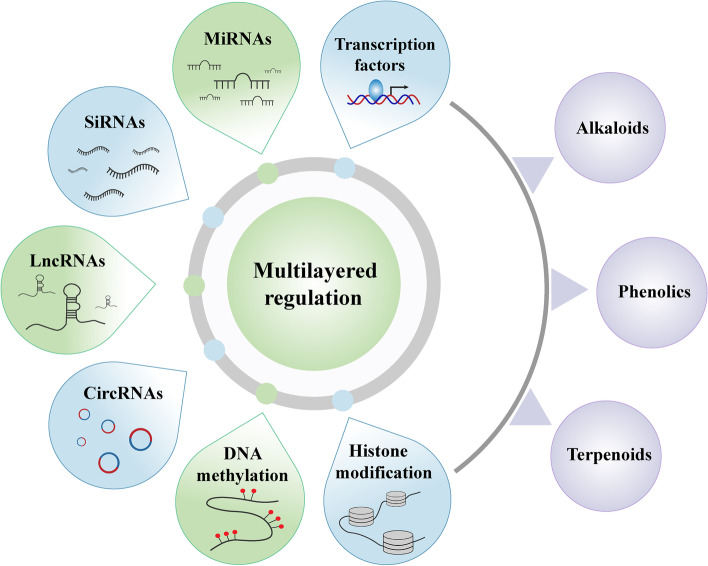

**Supplementary Information:**

The online version contains supplementary material available at 10.1186/s43897-023-00059-y.

## Introduction

**Fig. 1:**
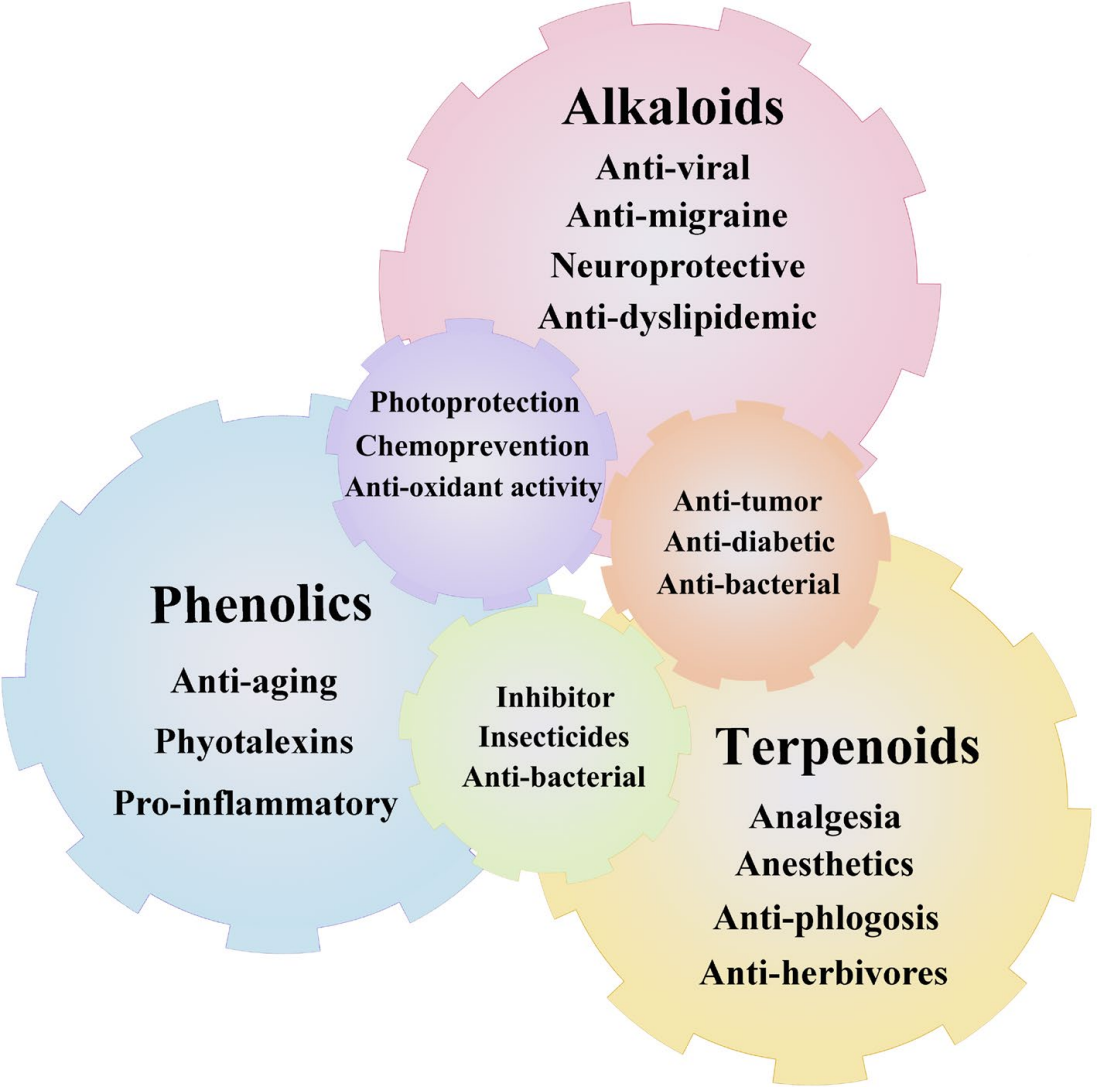
Physicochemical properties of secondary metabolites in medicinal plants. The abundance of these compounds, such as phenolics, terpenoids, and alkaloids, makes medicinal plants rich in nutraceutical and pharmaceutical properties. There are multiple health benefits in the bioactive compounds present in medicinal plants, including antidiabetic, anticancer, galactagogic, digestive, hepatoprotective, regulatory, and antioxidant properties, and work against anorexic, antilithogenic, and antipathogenic properties, and several other medicinal properties

**Fig. 2:**
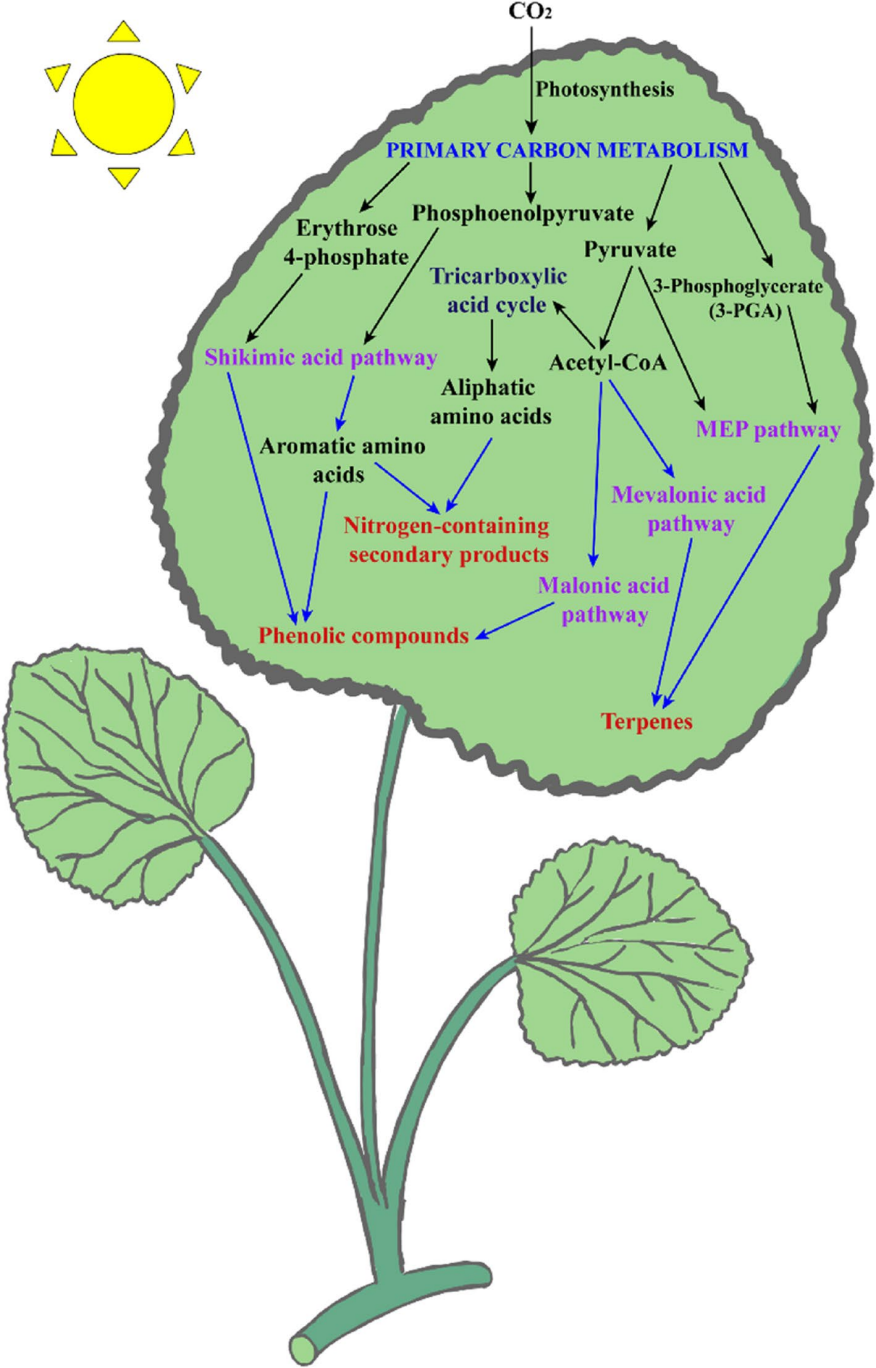
Schematic representation of the major pathway of secondary metabolite biosynthesis and its interrelationships with primary metabolites. Primary metabolites are interconnected with secondary metabolism by producing secondary metabolite compounds through the biosynthetic pathway. Types of biosynthetic pathways in plant secondary metabolism are shikimic acid (shikimate), malonic acid (malonate), mevalonic acid (mevalonate), and methylerythritol-phosphate pathways. Plant secondary metabolites (SMs) are mainly divided into three different groups, including terpenes, phenolics and nitrogen-containing compounds, derived from photosynthesis as the primary carbon metabolism process through the four biosynthetic pathways

**Fig. 3:**
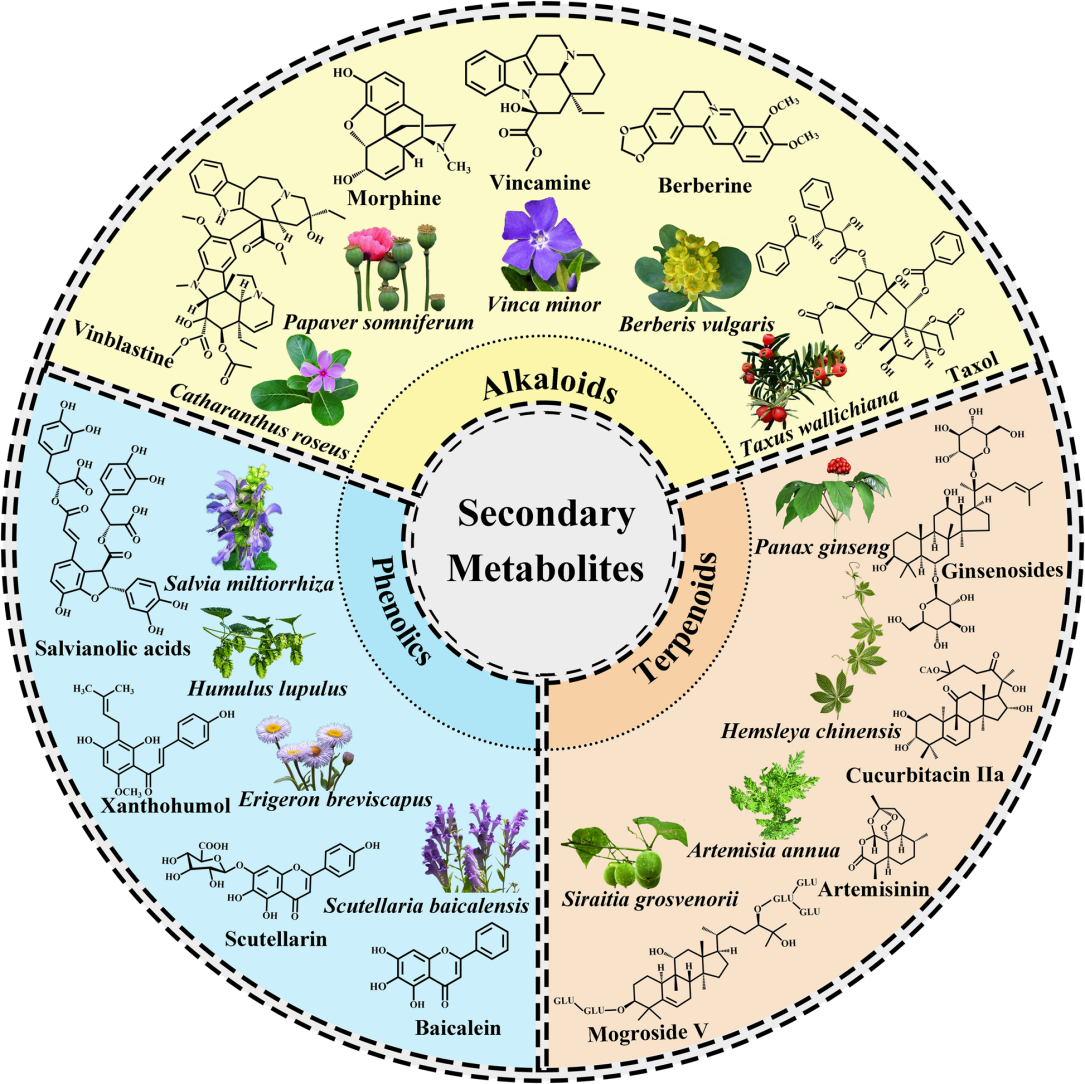
Chemical structure examples of secondary metabolites produced by medicinal plants. Plants yield a wide variety of active ingredients that are identified and isolated. Compounds are organized in accordance with their biosynthesis pathways. The group of phenolic compounds consists of (1) salvianolic acids from *Salvia Miltiorrhiza*; (2) xanthohumol from *Humulus lupulus*; (3) scutellarin from *Erigeron breviscapus*; and (4) baicalein from *Scutellaria baicalensis*. The group of terpenoid compounds consists of (1) ginsenosides from *Panax ginseng*; (2) cucurbitacin IIa from *Hemsleya chinensis*; (3) artemisinin from *Artemisia annua*; and (4) mogroside V from *Siraitia grosvenorii.* The group of alkaloid compounds consists of (1) vincristine from *Catharanthus roseus*; (2) morphine from *Papaver somniferum*; (3) vincamine from *Vinca minor*; (4) berberine from *Berberis vulgaris*; and (5) taxol from *Taxus wallichiana*

Plants as living chemical factories synthesize a wide variety of secondary metabolites (SMs), which are not directly involved in the primary processes of growth and reproduction, but often have important ecological functions. The majority of plants share the same fundamental biosynthetic pathways, with most primary metabolites present in every tissue. The maintenance of this metabolic core has resulted in a limited number of metabolic frameworks. Frequent modifications, such as glycosylation, methylation, acylation and phosphorylation, as well as a few chemical changes due to tailored enzymes, generate a variety of modifications in basic structures (Morreel et al. 2014). The different chemical constituents in medicinal plants provide biological activities that can benefit human health through the pharmaceutical and food industries, but they also represent significant value in the perfume, agrochemical, and cosmetic industries (Hassan 2013) (Fig. 1). In terms of their biosynthenic pathways (Fig. 2), SMs can be classified into three main groups: phenolic compounds synthesized in the shikimate pathway, terpenes synthesized in the mevalonic pathway, and nitrogen-containing compounds synthesized in the tricarboxylic acid cycle pathway (Jamwal et al. 2018; Sanchita and Sharma 2018) (Fig. 3). However, the synthesis of these metabolites poses challenges, such as their low concentrations in plant parts. Thus, researchers are trying to find ways to improve their contents in medicinal plants. This can be done through the metabolic engineering of plants to either enhance the production of desirable compounds or reduce the production of undesirable compounds.

Scientists’ understanding of how secondary metabolites are regulated in medicinal plants, including transcriptional regulation, has been growing. Transcription factors (TFs) play a role in plant defense by detecting stress signals and directing downstream defense gene expression. However, the molecular mechanisms regulating SMs accumulation in medicinal plants without affecting their normal growth and development are not well understood (Zheng et al. 2023). Similarly, plant survival, durability, and productivity are all dependent on increased synthesis, known as elicitation, of secondary metabolites. Various biotic (fungi, bacteria, etc.) and abiotic (exogenous hormones) elicitors have been used to enhance the production of secondary metabolites in plants to protect them from stress stimuli (Jan et al. 2021). Recently, epigenetic regulation of secondary metabolites in medicinal plants has gained increased attention. Epigenetics refers to any non-genetic heritable molecular modification of the genome that may alter gene expression (Meyer et al. 2018). Additionally, epigenetic modifications influence and regulate many aspects of plant development and physiology. DNA methylation at cytosine positions has been shown to affect gene expression, transposon activity, and chromosome interactions (Zhang et al. 2018a), modulating plant development and responding to environmental clues (Zhang et al. 2018a; Vidalis et al. 2016). It has recently been shown that changes in DNA methylation patterns may affect gene expression in *cis* and in *trans* possibly via small RNAs affecting primary and specialized metabolic pathways in *Arabidopsis thaliana* (Kooke et al. 2019). In addition, histone and DNA modifications are likely to shape relationships between cell metabolites and the corresponding gene expression (Leung et al. 2020). Specialized metabolic pathways in plants undergo both developmental and environmental regulation, and additional epigenetic control has been proposed. Previous studies have shown that inhibitors of DNA methylation are able to increase phenolic product biosynthesis in *Salvia miltiorrhiza* (Bunge) hairy root cultures (Yang et al. 2018).

When considering the genetic regulation of secondary metabolites, it is important to note that the genome is the “hardware” they are born with. Their epigenetic modifications to DNA and associated proteins are the “software” influencing gene expression. Here we discuss recent advances in multilayer regulation related to secondary metabolism in medicinal plants, consider epigenetic enzymes including “writers”, “erasers” and “readers”, and then demonstrate the essential roles of epigenetic regulation of SMs in medicinal plants. We also propose a multilayer understanding of genetic and epigenetic regulation and their roles in regulating gene expression and SM accumulation in medicinal plants.

## Biosynthesis of secondary metabolites in medicinal plants

SMs in medicinal plants are generated by different metabolic pathways. Different environments and temperatures affect the quantity and quality of these compounds. Moreover, the biosynthesis of SMs is highly interconnected/interrelated with the primary metabolism inside the plant cell. Terpenes are synthesized in two major pathways: mevalonic-acid (MVA) pathways and 2-C-methylerythritol 4-phosphate (MEP) pathways, the latter of which occurs in the plastid. In *Escherichia coli*, the MEP pathway was initially elucidated, and subsequently plant homologues have been characterized through biochemical and genomic methods (Rodriguez-Concepcion and Boronat 2002). Products of glycolysis such as pyruvate or acetyl-CoA are responsible for the synthesis of isopentenyl pyrophosphate (IPP) and dimethylallyl pyrophosphate (DMAPP) which act as universal precursors for all terpenoids localized in various cellular compartments (Nagegowda et al. 2010).

In plants, phenolic compounds are produced via the shikimic acid and malonic acid pathways (Ghasemzadeh and Jaafar 2011). There has also been evidence of the malonic acid pathway in fungi and bacteria for the synthesis of phenolic compounds (Cheynier et al. 2013). In response to various stress constraints, phenylalanine ammonia lyase (PAL) and chalcone synthase (CHS) regulate phenol synthesis (Sharma et al. 2019). Nitrogen-containing SMs contain nitrogen molecules in their structure, and amino acids such as lysine, tyrosine and tryptophan act as precursors in their biosynthesis. Researchers have found that SMs are varied and complex in different parts of medicinal plants, and that they may be synthesized via special regulatory pathways and transport routes in certain organs. By forming and accumulating precursors, SMs can be regulated at various levels, starting with the transport and metabolism of extracellular nutrients. Therefore, it is imperative to investigate changes found in the expression patterns of genes involved in secondary metabolite biosynthesis in medicinal plants. Genetic regulation of secondary metabolism refers to the control of the production and synthesis of secondary metabolites in an organism through genetic mechanisms. The regulation of secondary metabolite production can occur at different levels, including transcriptional regulation, post-transcriptional regulation, translational regulation, and post-translational regulation. Here we summarize the transcriptional regulation and post-transcriptional regulation of secondary metabolites in medicinal plants.

## Elucidation of transcriptional regulation of secondary metabolism

Transcriptional regulation via TFs leads to an alteration of the inducible synthesis of SMs and the transcription of biosynthetic genes at various levels. TFs are DNA binding proteins that attach to the promoter regions of target genes and change the rate of transcriptional initiation via RNA polymerases. Furthermore, the accumulation of SMs is controlled by TFs that integrate external and internal signals to regulate the expression of enzyme genes. A wide array of TFs affects the regulation of genes related to the SM biosynthesis pathways (Yang et al. 2012). The identification of TFs and investigation of their regulatory mechanisms in SM biosynthesis pathways has increased in recent decades. Here we present the TF families that act independently or cooperatively, to simultaneously regulate SMs in medicinal plants.

## bHLH family

The bHLH proteins (basic helix-loop-helix proteins) are one of the largest transcription factor families in plants, containing the highly conserved bHLH structural domain, by which HLH and basic regions are combined (Li et al. 2021). bHLH is a helix-loop-helix structure, containing 45 amino acids forming a dimerization motif that is essential for homodimerization or heterodimerization (Kavas et al. 2016; Mao et al. 2017; Xiang et al. 2015). Previous studies have shown that the 162 *bHLH* TFs in *A. thaliana* are divided into 21 subfamilies (Toledo-Ortiz et al. 2003), the 169 *bHLH* TFs in *Panax ginseng* can be classified into 24 subfamilies (Chu et al. 2018), the 167 *bHLH* TFs available in *Oryza sativa* can be classified into 22 subfamilies (Li et al. 2006), the 115 *bHLH* TFs identified in *Vitis davidii* can be classified into 25 subfamilies (Li et al. 2021), the 188 *bHLH* TFs in *Malus domestica* (Malus × domestica) can be classified into 18 subfamilies (Mao et al. 2017) and the 230 *bHLH* TFs in Chinese cabbage (*Brassica rapa ssp. pekinensis*) are classified into 24 subfamilies (Song et al. 2014). Currently, approximately 28,698 *bHLH* TFs have been identified in 166 species in the Plant Transcription Factor Bank (https://planttfdb.gao-lab.org/). Approximately 20 or more of these plants are medicinal plants.

Many bHLHs have been shown to influence plant growth, development, and responses to abiotic stresses (Zhou et al. 2020), and exert an impact on secondary metabolites such as alkaloids, terpenoids and flavonoids in medicinal plants (Heim et al. 2003; Zhou et al. 2020). For example, *CjbHLH1* regulates the biosynthesis of quinoline alkaloids in *Coptis japonica* (Yamada et al. 2011). Another bHLH transcription factor, MYC, is well-studied and involved in several signaling pathways (such as biotic, abiotic, and developmental responses). *Catharanthus roseus* contains two MYC TFs, *CrMYC1* and *CrMYC2* (Chatel et al. 2003; Zhang et al. 2011a, b). *CrMYC1* is involved in the regulation of methyl jasmonate in *C. roseus*, and *CrMYC2* is an early methyl jasmonate response factor, that is involved in the expression of a series of terpenoid indole alkaloid (TIA) synthase genes by regulating the expression of *ORCA* genes.

**Fig. 4:**
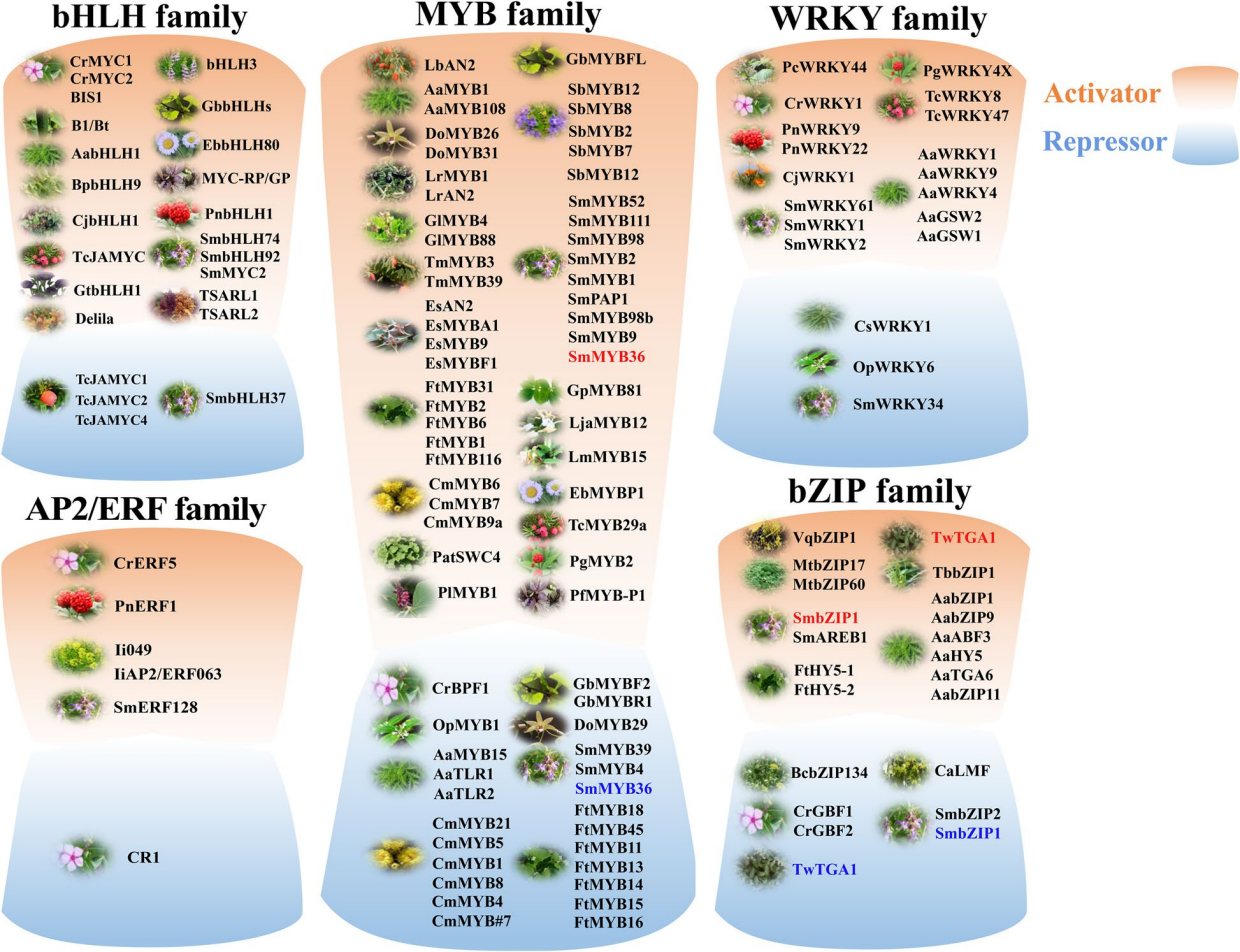
Transcriptional regulation of secondary metabolites biosynthesis in medicinal plants. The pathway-specific functions of transcription factors are illustrated, with activators grouped in red and repressors in blue. Different medicinal plants contain a number of TFs involved in the biosynthesis pathway. An overview of a transcriptional network that is involved in SM biosynthesis in medicinal plants is presented in this review. A large number of positive TFs is identified, but the number of negative TFs is limited. Negative regulatory TFs also play a critical role in establishing the dynamic balance in plant secondary metabolism

Another role of bHLHs is the regulation of flavonoid synthesis. For example, MYC-RP/GP in *Perilla frutescens* (Gong et al. 1999), *GtbHLH1* in gentian (Nakatsuka et al. 2008), *ScbHLH17* in *Senecio cruentus* (Li et al. 2020), etc., are all bHLH TFs that regulate anthocyanin synthesis. Furthermore, when *EbbHLH80* from *Erigeron breviscapus* was heterologously expressed in tobacco, flavonoid levels were significantly increased (Gao et al. 2022). Furthermore, homodimers or heterodimers are the most common forms of bHLH-type TFs (Goossens et al. 2017). A heterodimer formed between SmbHLH60 and SmMYC2 that regulates the biosynthesis of phenolic acid and anthocyanins antagonistically (Liu et al. 2022). Terpenoids are defense compounds in most medicinal plants that provide protection against pathogens or predation by herbivores, and as the synthesis pathways of many terpenoids have been discovered, some *bHLH* TFs regulating terpenoid synthesis pathways have received attention. For example, *TcJAMYC* was found to be involved in the biosynthesis of taxol in *Taxus* (Nims et al. 2015). Furthermore, Shang et al. (2014) identified two bHLH TFs, *B1* (bitter leaf) and *Bt* (bitter fruit), that regulate the synthesis of cucurbitacin. Similarly, bHLH iridoid synthesis 1 (*BIS1*) is involved in the monoterpene (iridoid) branch of the monoterpene indole alkaloid pathway (Van Moerkercke et al. 2015). In *Panax notoginseng*, *PnbHLH1* improves the biosynthesis of triterpenoids by interacting with the E-box core sequence in the promoter region of the target gene (Zhang et al. 2017). A gene-specific expression pattern and methyl jasmonate (MeJA)-induced upregulated expression pattern led to the identification of six bHLH TFs (*PGbHLH*s) involved in ginsenoside synthesis (Chu et al. 2018). Additionally, Mertens et al. (2016) found that *TSAR1* and *TSAR2* from *Medicago truncatula* increased the accumulation of triterpene saponins in response to jasmonic acid. *TSAR3* significantly promotes hemolytic saponin production (Ribeiro et al. 2020). *TSARL1* and *TSARL2* were found in quinoa with *TSAR1* and *TSAR2*, and were found to regulate the expression of genes related to triterpene synthesis (Jarvis et al. 2017). *bHLH3* is involved in the biosynthesis of triterpene saponins in licorice (*Glycyrrhiza glabra*) (Tamura et al. 2018). Additionally, *SmbHLH37*, *SmbHLH74*, and *SmbHLH92* perfectly fit the accumulation pattern of tanshinone (Du et al. 2018; Zhang et al. 2015a, b). Information on bHLH factors involved in regulating SMs in medicinal plants is shown in Fig. 4 and Supplementary Table 1. Currently, relatively few bHLH transcription factors have been identified in medicinal plants, and additional studies in this area could help to reveal the secondary metabolic pathways in medicinal plants.

## MYB family

Myeloblastosis (MYB) proteins comprise a superfamily of TFs that participate in the biosynthesis of SMs and in various biological processes in plants, such as growth, reproduction, and stress responses. Their domain consists of 1-4 repeats, each of which contains 50-53 amino acids encoding three α-helices (Martin and Paz-Ares 1997). In terms of the number and location of the MYB domains, they can be divided into four categories: 1R (R1/2, R3-MYB), 2R (R2R3-MYB), 3R (R1R2R3-MYB), and 4R (R1R2R2R1/2MYB) (Dubos et al. 2010). R2R3-MYB, the major class of MYB factors, has been divided into 28 subgroups based on the motifs in their most C-terminal MYB domain (Stracke et al. 2001). Since the first plant MYB was found in *Zea mays* (Paz-Ares et al. 1987), approximately 22,032 MYB and 15,369 MYB-related sequences have been identified in 165 species in the Plant Transcription Factor Database (http://planttfdb.gao-lab.org/). Among these plants, 21 species were medicinal plants (Thakur and Vasudev 2022).

MYBs have been shown to perform multiple important biological functions, such as regulating primary and secondary metabolism, controlling the circadian rhythm, determining cell identity and fate, and transducing hormonal signals (Cao et al. 2020; Dubos et al. 2010; Ambawat et al. 2013). The biosynthesis of secondary metabolites is a survival tactic for plants to respond to environmental changes (Jan et al. 2021). Previous studies on MYB regulation of secondary metabolism have mainly focused on nonmedicinal plants. For example, R2R3-MYB factor subgroups 4-7 were reported to control phenylpropanoid biosynthesis in *A. thaliana* (Liu et al. 2015; Deng and Lu 2017): AtMYB32 from subgroup 4 repressed monolignol biosynthesis; AtMYB123/TT2 from subgroup 5 promoted proanthocyanidin biosynthesis (Baudry et al. 2004); AtMYB75/PAP1 (production of anthocyanin pigment 1), AtMYB90/PAP2, AtMYB113, and AtMYB114 belonged to subgroup 6 and regulated anthocyanin biosynthesis (Gonzalez et al. 2008; Xie et al. 2006; Borevitz et al. 2000); and AtMYB11, AtMYB12, and AtMYB111 in subgroup 7 had a function in the regulation of flavonol biosynthesis (Mehrtens et al. 2005; Stracke et al. 2007; Stracke et al. 2010).

With the development and utilization of active SMs in medicinal plants, more research has shifted to medicinal plants for the biosynthesis and regulation of secondary metabolites. Currently, the regulation of SMs by MYBs in medicinal plants focuses on the biosynthesis of flavonoids, phenolic acids, and terpenoids. In *Scutellaria baicalensis*, *SbMYB12* was found to activate the expression of the *SbCCL7-4*, *SbCHI-2*, and *SbF6H-1* genes and positively regulate the generation of baicalin and wogonoside (Wang et al. 2022a). Additionally, the *GlMYB4* and *GlMYB88* from *Glycyrrhiza uralensis* could positively regulate flavonoid synthesis in licorice cells induced by MeJA (Li et al. 2020). *Ginkgo biloba* is another medicinal plant rich in flavonoids in which *GbMYBF2* and *GbMYBFL* play opposite roles in regulating flavonoid biosynthesis as a repressor and an activator, respectively (Xu et al. 2014; Zhang et al. 2018b). In a series of research studies on *Epimedium sagittatum*, Huang et al. identified several MYB proteins that promote flavonoid biosynthesis, such as *EsMYB9*, *EsMYBA1*, *EsAN2*, and *EsMYBF1 *(Huang et al. 2013; Huang et al. 2016a; Huang et al. 2016b; Huang et al. 2017). Information on MYB TFs involved in flavonoid synthesis in other medicinal plants is shown in Fig. 4 and Supplementary Table 2. Two MYB proteins (SmMYB1 and SmMYB2) from *Salvia miltiorrhiza* were proven to upregulate the expression of the *CYP98A14* gene and significantly promote salvianolic acid accumulation (Zhou et al. 2021; Deng et al. 2020a, b), and *SmMYB9b* and *SmMYB98b* positively regulate tanshinone biosynthesis in the hairy roots (Liu et al. 2020). *SmMYB4*, on the other hand, functions as a repressor in the biosynthesis of phenolic acids and tanshinones (Tian et al. 2022). An R2R3 type MYB transcription factor *LmMYB15* gene from *Lonicera macranthoides* was isolated and characterized by Tang et al. (2021), who found that it might facilitate chlorogenic acid biosynthesis via direct transcriptional activation of the *4CL* gene. The transcriptional regulation of terpenoids is mainly found in monoterpene, sesquiterpene, and triterpene saponins. As a kind of sesquiterpene lactone with significant antimalarial effects, artemisinin is synthesized and stored in the glandular trichome of *Artemisia annua* leaves. *AaMYB1* could positively regulate trichome initiation and artemisinin biosynthesis, while *AaMYB15* led to a significant decline in the expression levels of the *AaADS*, *AaCYP*, *AaDBR2*, and *AaALDH1* genes and decreased the artemisinin contents in *A. annua* (Wu et al. 2021). *AaTLR1* and *AaTLR2* also reduced artemisinin levels by inhibiting trichome development (Lv et al. 2022). In *Panax ginseng*, *PgMYB2* was reported to improve ginsenoside production by promoting *PgDDS* gene expression (Liu et al. 2019a). Similarly, *PnMYB2* isolated from *Panax notoginseng* was considered likely to regulate the biosynthesis of ginsenoside, but its specific functions were still unclear (Xia et al. 2022). *GpMYB81* can bind to the promoters of the *GpFPS1* and *GpCHS* genes and activate their expression, acting as a “dual-function” regulator of gypenoside and flavonol biosynthesis in *Gynostemma pentaphyllum* (Huang et al. 2022). Furthermore, *OpMYB1* from *Ophiorrhiza pumila* and *CrBPF1* from *Catharanthus roseus* had the function of regulating alkaloids biosynthesis (Rohani et al. 2016). *EbMYBP1* from *E.breviscapus* was a activator involved in the regulation of flavonoid accumulation (Zhao et al. 2022). These results will be helpful for further research on the complex regulatory mechanism of secondary metabolite formation in medicinal plants.

## WRKY family

WRKY transcription factors play a significant role in seed germination, seed dormancy, and response to stress in plants (Rushton et al. 2010). The first WRKY gene was cloned from sweet potato (Ishiguro and Nakamura 1994), and a large number of WRKY genes have been isolated and identified from medicinal plants such as *A. annua* (Ma et al. 2009), *P. ginseng* (Di et al. 2021), *P. notoginseng* (Zheng et al. 2022), *Ophiorrhiza pumila* (Wang et al. 2022), *Artemisia argyi* (Zhang et al. 2022), and *Cannabis sativa* (Liu et al. 2021). WRKY TFs have significant structural characteristics; their structure contains 1-2 WRKY domains, which are DNA binding domains composed of approximately 60 highly conserved amino acid residues. WRKYGQK, a heptapeptide located at the N-terminal, is the core sequence, and the sequence located at the C-terminal consists of C2H2 (C-X_4-5_-C-X_22–23_-H-X-H) or C2HC (C-X_7_-C-X_23_-H-X-C) zinc finger structures. Based on their DBD and zinc finger motifs, WRKYs are classified into groups I, II, and III, and group II is further divided into IIa, IIb, IIc, IId and IIe corresponding to the primary amino acid sequence (Eulgem 2000; Jiang et al. 2017). Both C2H2 and C2HC motifs are necessary for WRKY proteins to interact with W-box (TTGACT/C) cis-elements in many gene promoters, as they can activate or inhibit transcription by recognizing and binding the W-box (TTGACT/C) in its target gene, and recognizing its own promoter or other target genes to achieve regulatory effects (Bakshi and Oelmüller 2014). The multiple W-boxes of the WRKY gene indicate that self-regulation and cross-regulation are the characteristics of the WRKY TF regulatory network.

WRKY TFs respond to pathogens and defense-related plant hormones such as SA or JA, which means that the WRKY gene family plays an important role in plant immunity (Wani et al. 2021). WRKY proteins play a vital role in mediating the expression of many genes by binding to W-box elements in the promoter region during pathogen infection, which correlates with the modulation of JA and SA signaling pathways. Methyl jasmonate (MeJA) enhances the resistance of *P. notoginseng* to *F. solani*, which indicates that JA signaling appears to play a vital role in *P. notoginseng* responses to *F. solani* infection (Liu et al. 2019b). The expression levels of WRKY genes increased in response to MeJA treatment and subsequent *F. solani* infection. *PnWRKY9* recombinant protein was observed to bind specifically to the W-box sequence in the promoter of a JA-responsive and *F. solani* resistance-related defensin gene (*PnDEFL1*). The overexpression of *PnWRKY9* in tobacco considerably increased the resistance to *F. solani*, whereas an RNAi-mediated decrease in the *PnWRKY9* expression level in *P. notoginseng* leaves increased the susceptibility of tobacco to *F. solani* (Zheng et al. 2022). *PnWRKY22* acts as a hub gene in the defense response of resistance to root rot. Transiently overexpressing *PnWRKY22* increased salicylic acid levels in *P. notoginseng* leaves (Ning et al. 2021).

Similar to many other TFs, WRKYs regulate secondary metabolism, including phenylpropanoid, terpene, and alkaloid metabolism (Schluttenhofer and Yuan 2015). Information on WRKY TFs involved in SM synthesis in other medicinal plants is shown in Fig. 4 and Supplementary Table 3. Moreover, WRKY TFs regulate a diverse array of specialized plant metabolites that have diverse biological functions. Artemisinin, a sesquiterpene lactone widely used in drugs to fight malaria, was discovered in *Artemisia annua*. Previous research recognized *AaWRKY1* in the regulation of artemisinin biosynthesis and showed that amorpha-4,11-diene synthase (ADS) is a target gene of *AaWRKY1* (Ma et al. 2009). In addition, glandular trichome-specific WRKY 1 *AaGSW1* directly binds to W-boxes in the promoters of *AaCYP71AV1* and *AaORA*, and positively promotes artemisinin biosynthesis in *Artemisia annua* (Chen et al. 2017). Further study showed that *AaWRKY9* positively regulates artemisinin biosynthesis by directly binding to the promoters of *AaDBR2* and *AaGSW1* (Fu et al. 2021). The genus *Panax*, commonly known as ginseng, in the family Araliaceae contains traditional medicinal plants used around the world and has been used to produce ginsenosides. A total of 137 *PgWRKY* genes were identified from the ginseng genome. Coexpression analysis identified 11 *PgWRKYs* that may have a potential regulatory role in the process of ginsenoside biosynthesis (Di et al. 2021). *PgWRKY4X* binds to the W-box of the squalene epoxidase (*PgSE*) promoter. Overexpression of *PgWRKY4X* could significantly enhance ginsenoside accumulation in *P. ginseng* transgenic cells. Moreover, the transcriptional levels of *PgWRKYs* were analyzed and the correlation analysis showed that *GPS*, *SS*, *CYP716A47*, *CYP716A53v2*, *UGT74AE2*, *UGT94Q2*, *PgWRKY1*, *PgWRKY3*, and *PgWRKY8* were significantly correlated with total ginsenoside content (Yao et al. 2020). *Ophiorrhiza pumila* is a medicinal plant model for the study of the biosynthesis of camptothecin (CPT), which is a pentacyclic quinoline alkaloid widely used in anticancer drugs worldwide. Forty-six *OpWRKY* genes were identified in the *O. pumila* genome. Overexpression of *OpWRKY6* significantly reduced the accumulation of camptothecin compared with the control. Conversely, camptothecin accumulation increased in *OpWRKY6* knockout lines (Wang et al. 2022). Cannabinoids are important secondary metabolites present in *Cannabis sativa*. *CsWRKY1* is an opposite regulator as delta-9-tetrahydrocannabinolic acid synthase expression (THCAS) (Liu et al. 2021)

## AP2/ERF family

The AP2/ERF (APETALA2/Ethylene response factor) transcription factor family is primarily responsible for regulating the stress response of plants (Shukla et al. 2006; Navarro et al. 2009; Tang et al. 2007; Liu et al. 2011), regulating the growth and development of plants (Feng et al. 2020), and participating in the regulation of some secondary metabolic pathways in medicinal plants (Xiao et al. 2023; Menke et al. 1999; Yu et al. 2012). AP2/ERF sequences contain at least one AP2 domain composed of approximately 60 amino acids. AP2/ERF has five subfamilies based on their cis elements: ERF (ethylene-responsive factor), AP2 (APETALA2), DREB/CRT (dehydration-responsive element binding factor), RAV (related to ABI3/VP1), and Soloist. The ERF subfamily contains an AP2/ERF domain (recognition motif: AGCCGCC), whose main function is to regulate the response of plants to partial stress and secondary metabolic pathways. The AP2 subfamily has two highly similar AP2/ERF domains (recognition motif: GCAC (A/G) N (A/T) TCCC (A/G) ANG (C/T)), whose main function is to regulate plant growth and development. The DREB/CRT subfamily contains only one AP2/ERF domain (recognition motif: GCAC (A/G) N (A/T) TCCC (A/G) ANG (C/T)), whose main function is to regulate the response of plants to stress. The difference between the DREB/CRT subfamily and the ERF subfamily is that the 14th and 19th amino acids of the two subfamilies are different. The RAV subfamily contains an AP2+B3 domain (recognition motif: CAACA). Its main function is to regulate plant growth and development and response to partial stress. The Soloist subfamily has an AP2 domain with low homology with other subfamilies, and its main function is to regulate the response of plants to partial stress.

ERF transcription factors play an important role in the regulation of secondary metabolism pathways in plants that produce compounds of high pharmaceutical importance. For example, the expression level of some *ERF* genes in ginseng will be affected under cold stress, and the *PgERF* gene family is responsive to MeJA (Chen et al. 2020). *CrERF5* in *C. roseus* responds to ethylene and JA signals. Information on AP2/ERF TFs involved in SM synthesis in other medicinal plants is shown in Fig. 4 and Supplementary Table 4. Overexpression of *CrERF5* in the petals of *C. roseus* will lead to a significant increase in the expression level of key genes upstream and downstream of the biosynthesis of MIAs (monoterpenoid indole alkaloids), while the silencing of *CrERF5* will lead to a decrease in the expression level of key genes, indicating that *CrERF5* will affect the accumulation level of MIAs by regulating the genes in the MIA biosynthesis pathway (Pan et al. 2019). *C. roseus* is considered a model plant for studying the biosynthesis of TIAs. In the leaves of *C. roseus* treated with methyl jasmonate (MeJA), a candidate gene *CR1* that may participate in the regulation of TIA biosynthesis in *C. roseus* was screened by RNA-seq combined with phylogenetic analysis. Silencing *CR1* increased the accumulation of vindoline and serpentine in *C. roseus* (Liu et al. 2017). In addition, the AP2/ERF families are also involved in the synthesis of triterpene saponins in plants. *PnERF1* contains a conserved AP2 domain, which may promote the biosynthesis of *P. notoginseng* saponins by regulating the expression level of key enzyme genes in the biosynthesis pathway of triterpenoid saponins (Deng et al. 2017). Two genes (*SmERF128* and *SmERF152*) in *S. miliorrhiza* regulate the biosynthesis of tanshinone, belonging to the ERF-B3 subgroup (Ji et al. 2016). Furthermore, *SmERF128* from *S. miltiorrhiza* positively regulates tanshinone biosynthesis by activating the expression of *SmCPS1*, *SmKSL1* and *SmCYP76AH1* (Zhang et al. 2019). Additionally, the antiviral properties of lignans, such as lariciresinol and its derivatives, have been identified in *Isatis indigotica*. Evidence suggests that the AP2/ERF family might contribute to lignan biosynthesis in *I. indigotica.* Some highly expressed *IiAP2/ERF018*, *IiAP2/ERF054* and *IiAP2/ERF073* in the root may be involved in the regulation of the development and lignan biosynthesis of *I. indigotica* roots. Additionally, the expression levels of some ERF TFs (*IiAP2/ERF026*, *IiAP2/ERF054*, *IiAP2/ERF081* and *IiAP2/ERF090*) in *I. indigotica* changed after NaCl and PEG treatment. It is suggested that some *ERF* genes in *I. indigotica* can respond to abiotic stress (Xiao et al. 2023). The transcription factor *Ii049* of the AP2/ERF family in *I. indigotica* Fort. could be a positive regulatory effector, controlling lignan biosynthesis by regulating the genes involved in lignan biosynthesis and regulating SA biosynthesis, thus inducing lignan accumulation (Ma et al. 2017).

Several studies have reported that the AP2/ERF family plays various roles in plant development, stress responses and secondary metabolism in many plant species (Licausi et al. 2013). Most of the AP2/ERF TFs in the Chinese medicinal plant *T. hemsleyanum* show a positive response to chilling stress (Xie et al. 2022). Under drought stress, four AP2/ERF members (*MaERF008*, *MaERF037*, *MaERF054* and *MaERF058*) from *Melilotus albus* were upregulated, indicating that they had a certain drought-tolerant function (Phukan et al. 2018). Mentha RAP2-4 is a positive regulator of waterlogging resistance, drought resistance and salt tolerance (Phukan et al. 2018). Some *HpERFs* in *H.perforatum* have the ability to cope with low temperature, SA and osmotic stress (Zhang et al. 2021). Heterologous expression of the *DcAP2ERF#96* gene from *D.catenatum* Lindl. in *A. thaliana* resulted in significant repression of multiple ABA downstream genes, including *P5CS1* and *RD29A*, and revealed that *DcAP2ERF#96* is involved in the biological function of ABA signaling (Han et al. 2022). Both *AgDREB1* and *AgDREB2* in celery (*Apium graveolens* L.) can respond to low temperatures. Overexpressing *AgDREB2* in *A. thaliana* has higher stress tolerance than *A. thaliana* with *AgDREB1*-OE under cold, salt and drought treatments, but the tolerance is reversed under ABA treatment (Li et al. 2019a). Some AP2/ERF members in *D.longan Lour* regulate the early SE and development process of longan seeds, roots and flowers, and respond to MeJA, SA, ABA, 2,4-D and other exogenous hormones (Zhang et al. 2020).

## bZIP family

bZIP (basic leucine zipper motif) TFs are one of the most abundant and conserved gene families in eukaryotes (Nijhawan et al. 2008). bZIP TFs are named for their common bZIP conserved domain (Dröge-Laser et al. 2018). The bZIP structure contains 60 to 80 amino acids and is composed of two parts, a highly conserved DNA-binding basic composed of 20 amino acids and a relatively diversified leucine zipper region (Talanian et al. 1990). The basic amino acid region is located at the C-terminal region and through a fixed N-x7-R/K structure for sequence-specific DNA binding. The leucine zipper region is located at the N-terminal region, which consists of several heptapeptide repeats or hydrophobic amino acid residues, such as methionine, isoleucine, valine, etc. According to the similarity of 78 bZIP TFs in *A. thaliana* basic regions and other conserved regions, *AtbZIP*s were divided into 13 subfamilies including A~K, M and S (Dröge-Laser and Weiste 2018). Different subfamilies are named for their functions. For example, subfamily A plays a central role in ABA signal transduction and can respond to cis-elements by directly binding to ABA (ABRE; ACGTGG/TC) to regulate the expression of target genes (Ali et al. 2016). Since their discovery in model plants such as *A. thaliana* and rice, approximately 15,498 bZIP sequences have been identified in 165 species in the Plant Transcription Factor Database (http://planttfdb.gao-lab.org/).bZIP TFs play an important role in plant signal transduction (Hossain et al. 2010), biological and abiotic stress responses (Ying et al. 2012), regulation of growth and development (Alonso et al. 2009), and biosynthesis of secondary metabolites (Zhang et al. 2015a, b). bZIP TFs *HY5* and *HYH* in *A. thaliana* regulate anthocyanin synthesis (Zhang et al. 2011a, b). *DkbZIP5* in *Diospyros kaki* is involved in the ABA signaling response, and overexpression of *DkbZIP5* can upregulate the expression of *DkMYB4*, thus promoting the accumulation of proanthocyanidins (Akagi et al. 2012). Moreover, *SiHY5* in *Solanum lycopersicum* as the downstream gene of *CRY1a* can directly recognize and bind G-box and ACE elements in the promoters of the anthocyanin biosynthesis genes *CHS1*, *CHS2*, and *DFR* and activate their transcription, thus promoting anthocyanin biosynthesis (Liu et al. 2018). *TbbZIP1* in *T. brevicorniculatum* regulates the transcription of the *TbSRPP1* gene in the ABA signal transduction pathway, thereby affecting the synthesis of natural rubber (Fricke et al. 2013). Both *PgbZIP16* and *PgbZIP34* identified in *Punica granatum* can promote the accumulation of anthocyanins in tobacco leaves (Wang et al. 2022). By knocking out an allele of *VvbZIP36* using the CRISPR/Cas9 technique, a series of anthocyanin biosynthesis genes were activated in *VvbZIP36* knockout plants, leading to the accumulation of related metabolites (Tu et al. 2022).

Previous studies related to the regulation of bZIP TFs on secondary metabolism in medicinal plants have mainly focused on the biosynthesis of terpenoids, flavonoids and alkaloids. Information on bZIP TFs involved in SM synthesis in other medicinal plants is shown in Fig. 4 and Supplementary Table 5. For example, ABA induction could promote the accumulation of artemisinin, and the expression of *AabZIP1* in *Artemisia annua* was significantly increased under ABA induction. Studies have shown that AabZIP1 can bind to the ABA responsive element (ABRE) in the promoter sequence of ADS and CYP71AV1, a key enzyme gene for artemisinin synthesis, as well as activate the expression of *ADS* and *CYP71AV1* and positively regulate artemisinin synthesis (Shu et al. 2022). *AabZIP9* can bind the cis-element in the ADS promoter and activate its expression to positively regulate artemisinin biosynthesis (Shen et al. 2019). Moreover, ABA-induced *AaABF3* TF, a member of subfamily A of *bZIP* TFs, can directly bind and activate the expression of *ALDH1*, a key gene for artemisinin biosynthesis, thereby regulating artemisinin biosynthesis (Zhong et al. 2018). *AaHY5*, a member of the H subfamily of *bZIP* TFs and the central regulator of light-dependent artemisinin biosynthesis, can directly bind to ubiquitin E3 ligase AaCOP1, activating the expression of *AaGSW1*, a gene related to artemisinin biosynthesis, and regulate artemisinin biosynthesis (Hao et al. 2019). Studies on *Bupleurum chinense bZIP* TFs showed that *BcbZIP134* may play a negative regulatory role in saikosaponin biosynthesis (Xu et al. 2019). In *S. miltiorrhiza*, *bZIP* TFs can affect root morphology by regulating tanshinone biosynthesis. *SmbZIP7* and *SmbZIP20* are coexpressed with *SmKSL1* and *SmCYP76AH1*, the key genes of the tanshinone biosynthesis pathway, which may regulate tanshinone biosynthesis by affecting the expression of the latter two genes (Zhang et al. 2018a, b, c). *SmAREB1* promotes salvianolic acid biosynthesis by positively regulating the expression of *SmPAL*, *SmTAT*, *SmRAS* and *SmHPPD* (Jia et al. 2017). *SmbZIP1* negatively regulates tanshinone biosynthesis. The content of tanshinone in hairy roots of *S. miltiorrhiza* overexpressing in *SmbZIP1* was lower than that of the control group, while the content of tanshinone was significantly increased with *SmbZIP1*-knockout transgenic strains (Deng et al. 2020a, b). In addition, SmbZIP1 can directly bind to the promoter of the C4H1 gene and activate its expression to promote salvianolic acid biosynthesis. *CaLMF* in *Camptothecin acuminata* negatively regulates the expression of camptothecin synthesis pathway genes *CaTDC1*, *CaG8O*, *CaCYC1* and *Ca7DLS*, thus inhibiting the biosynthesis of camptothecin (Chang et al. 2019). CrGBF1 and CrGBF2 in *C. roseus* can specifically bind to the G-box in the Str promoter, a key gene for the synthesis of terpenoid indole alkaloids, and inhibit the synthesis of terpenoid indole alkaloids by downregulating the expression of Str (Sibéril et al. 2001). Thus, the biological function of regulating the synthesis of bZIP TFs in regulating secondary metabolites can effectively improve the yield and quality of medicinal plants.

## NAC family

NAC TFs have been shown to play a role in plant growth, development, and stress tolerance. NAC TFs are named for the three proteins, NAM (no apical meristem), ATAF1-2 and CUC2 (cup-shaped cotyledon), that contain a similar DNA binding domain. NAC proteins appear to be widespread in plants (Ernst et al. 2004). *AaNAC1* has been identified in *A. annua* and induced by dehydration, cold, salicylic acid (SA) and methyl jasmonate (MJ). *AaNAC1* can potentially be used for improving the content of artemisinin and drought tolerance *in A. annua* (Lv et al. 2016)*.* When CPT biosynthesis and regulation were studied using a coexpression network, *OpNAC1* suppressed loganic acid O-methyltransferase (*OpLAMT*) expression and regulated camptothecin biosynthesis (Hao et al. 2023).

### Regulation of secondary metabolism by non-coding RNAs

Although transcription factors are the key transcription regulators of secondary metabolites, plant non-coding RNAs (ncRNAs) also contribute to the production of bioactive compounds. The ncRNA class comprises four types, including microRNAs (miRNAs), small interfering RNAs (siRNAs), long non-coding RNAs (lncRNAs), and circular RNAs (circRNAs), all of which play a key role in modulating plant-related genes responsible for secondary metabolite biosynthetic pathways. The role of regulatory ncRNAs in medicinal plants, especially miRNAs, has received extensive research attention during the last few years. Here, we summarize what is known about non-coding RNAs in medicinal plants and explain what role these genes play in producing bioactive compounds.

## MiRNAs

**Fig. 5:**
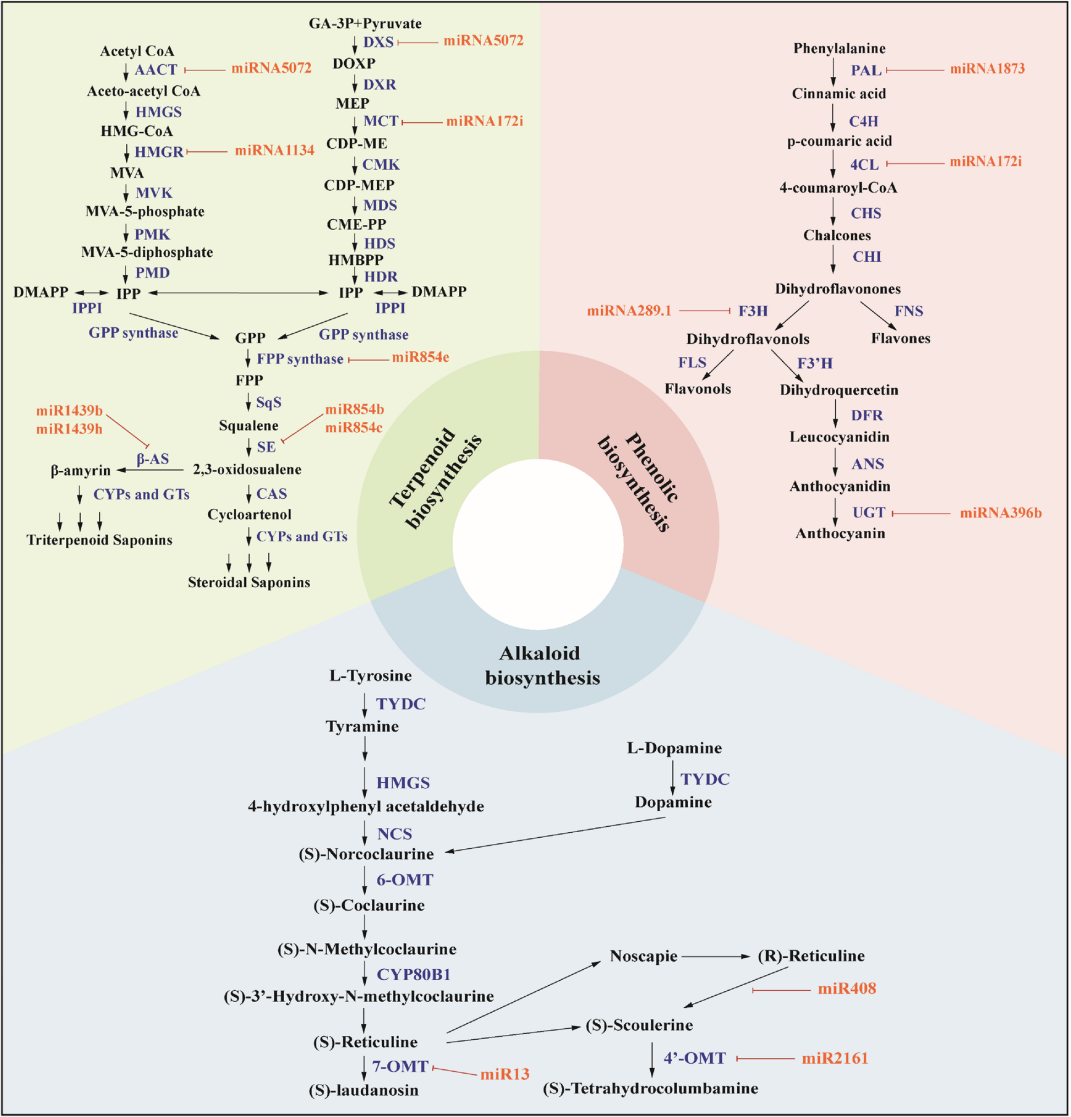
Regulatory mechanisms of miRNAs in terpenoid biosynthesis, phenolic biosynthesis, and alkaloid biosynthesis in medicinal plants. A large number of miRNAs participate in secondary metabolite synthesis by cleaving or repressing target mRNA. The modulating role of miRNA redirects secondary metabolites in plant cells for a specific biosynthetic pathway, thereby enhancing the production of therapeutic plant metabolites. The graph summarizes the current knowledge and understanding of miRNA and its role in the regulation, biosynthesis, and accumulation of secondary metabolites in plants, including alkaloids, terpenoids, and flavonoids. AACT, acetoacetyl CoA thiolase; HMGS, HMG-CoA synthase; HMGR, HMG-CoA reductase; MVK, mevalonate kinase; PMK, phosphomevalonate kinase; PMD, mevalonate diphosphate decarboxylase; IPPI (isopentenyl diphosphate isomerase); DXS, 1-deoxy-D-xylulose-5-phosphate synthase; DXR, 1-deoxy-D-xylulose 5-phosphate reductoisomerase; MCT, 2-C-methyl-D-erythritol 4-phosphate cytidylyltransferase; CMK, CDP-ME kinase; MDS, 2-C-methyl-D-erythritol 2,4-cyclodiphosphate synthase; HDS, (E)-4-hydroxy-3-methylbut-2-enyl diphosphate synthase; HDR, (E)-4-hydroxy-3-methylbut-2-enyl diphosphate reductase; FPPS, farnesyl diphosphate synthase; SQS, squalene synthase; SE, squalene epoxidase; β-AS, β-amyrin synthase; CAS, cycloartenol synthase; CYPs, cytochrome P450s; GTs, glandular trichomes. PAL, Phe ammonia-lyase; C4H, cinnamate-4-hydroxylase; 4CL, 4-coumaroyl:CoA-ligase; CHS, chalcone synthase; CHI, chalcone isomerase; F3H, flavanone 3-hydroxylase; FNS, flavones synthase; FLS, flavonol synthase; F3'H, flavonoid 3'-hydroxylase; DFR, dihydroflavonol 4-reductase; ANS, anthocyanidin synthase; UGT, UDP-glycosyltransferase; TYDC, tyrosine/dopa decarboxylase; HMGS, HMG-CoA synthase; NCS, norcoclaurine synthase; 6-OMT, 6-O-methyltransferase; CYP80B1, (S)-N-methylcoclaurine-3'-hydroxylase; 7-OMT, 7-O-methyltransferase; 4’-OMT, 4′-O- methyltransferase

MiRNAs are small non-coding RNA molecules involved in post-transcriptional gene regulation through their interaction with complementary sequences in the 3' untranslated region (3' UTR) of target mRNAs (Bartel 2009; Agarwal et al. 2015). When miRNAs interact with their target mRNAs, they can either degrade the mRNA or inhibit its translation, thus affecting gene expression. In medicinal plants, miRNA-mediated regulation contributes to the control of secondary metabolite synthesis, which involves controlling biosynthetic pathways and transcriptional regulators. Moreover, miRNAs target genes that produce alkaloids, flavonoids, and terpenoids, which could be important secondary metabolites (Fig. 5). For example, *miR13*, *miR408* and *miR2161* were recorded as potent miRNAs implicated to the regulation of alkaloid biosynthesis in *Papaver somniferum* (Boke et al. 2015). Several other authors have reported numerous miRNAs in plant alkaloid biosynthetic pathways in *Vinca minor* (Verma et al. 2020), *Rauvolfia serpentina* (Prakash et al. 2016), *C. roseus* I (Pani and Mahapatra 2013; Shen et al. 2017), and *Podophyllum hexandrum* (Biswas et al. 2016; Kumar et al. 2018).

Other miRNAs in the terpene biosynthesis pathway have been identified and their targets analyzed. Terpenoids or isoprenoids are a large and diverse class of volatile organic compounds. These chemicals are crucial to plant survival and evolution in a wide range of ecological regions, protecting molecules against biotic stresses, disseminating seeds, enhancing thermal tolerance, and attracting pollinators (Dudareva et al. 2006; Tetali 2019). Terpenoids also offer enormous potential in flavors, fragrances, pharmaceuticals, and biofuels (Tetali 2019). Since these compounds have a variety of applications, understanding their multiple functions would be helpful in regulating and manipulating their biosynthesis through genetic engineering (Abbas et al. 2017). The medicinal plant *P. kurroa* contains an enzyme called 3-deoxy-7-phosphoheptulonate synthase, whose mRNA is targeted by miR-4995, thereby regulating picroside synthesis (Vashisht et al. 2015). Saifi et al. (2015) further identified 11 miRNAs involved in the biosynthetic pathway of steviol glycosides in *Stevia rebaudiana*. Similarly, *miR7539*, *miR5021* and *miR1134* from *X. strumarium* might be involved in regulating terpenoid biosynthesis by targeting upstream terpenoid pathway genes (Fan et al. 2015). In addition, other research has supported that miRNA influences terpenoid biosynthesis in *Camellia sinensis* as well (Zhao et al. 2018). Five miRNAs including *miR2919*, *miR5251*, *miR838*, *miR5021*, and *miR5658* were determined to be related to the terpene biosynthetic pathway in *Ferula gummosa* via silico analysis (Najafabadi and Naghavi 2018). High-throughput sequencing and degradome analysis also identified the *Ginkgo biloba* miRNAs involved in the biosynthetic regulation of terpene trilactones (Ye et al. 2020). In the endangered medicinal plant *Podophyllum hexandrum Royle* (May apple), a previous study demonstrated that *miR1438* and *miR1873* regulate various metabolic pathways, especially the biosynthesis of secondary metabolites such as lignin and flavonoids via the caffeoyl-CoA O-methyl transferase and dihydroflavonol 4-reductase C genes, respectively (Biswas et al. 2016).

Plant miRNA expression is modulated by many environmental and genetic factors, such as stress, pathogen infection, and hormonal signals. MiRNAs play an important role in the regulation of secondary metabolite synthesis in medicinal plants, which can help develop strategies to increase the production of valuable medicinal compounds by understanding how these processes are regulated. In summary, miRNA-mediated regulation plays an important role in post-translational gene regulation in medicinal plants and contributes to the production of secondary metabolite synthesis.

## SiRNAs

SiRNAs, which are approximately 21–24 nucleotides in length, participate in abiotic stress response and secondary metabolism regulation in plants. Endogenous siRNAs in plants can be classified into subclasses based on their origins and biogenesis pathways, including transacting siRNAs (ta-siRNAs), natural antisense-derived siRNAs (nat-siRNAs), and heterochromatic siRNAs (hc-siRNAs) (Vazquez et al. 2010). In *Arabidopsis*, *OnSEN*, a copia-like retrotransposon, activates siRNA biogenesis mutants under heat stress by targeting *HSFA1* and *HSFA2* (Ito et al. 2011). Additionally, by targeting CHS genes such as CHS7 and CHS8 in *Glycine max*, primary siRNAs can then be generated, which suppress all CHS gene expression and inhibit flavonoid biosynthesis. Interestingly, this silencing mechanism is unique to the seed coat in *G. max*, not to all other organs/tissues (Tuteja et al. 2009). In other plants, such as *Petunia hybrids*, flavonoid and anthocyanin synthesis are regulated via siRNA (Morita et al. 2012).

## LncRNAs and circRNAs

LncRNAs are known to play an important role in the plant response to abiotic stress (Wang et al. 2017). Moreover, there has been some evidence showing that lncRNAs in medicinal plants were involved in the regulation of secondary metabolism. They were identified in species such as *S. miltiorrhiza* (Li et al. 2015a, b), *P. ginseng* (Wang et al. 2015), *D. purpurea* (Wu et al. 2012) and *G. sylvestre* (Ayachit et al. 2019). Additionally, circRNAs play an important role in plant development and response to abiotic and biotic factors. For example, overexpression of a circRNA (Vv-circATS1), which originated from glycerol-3-P acyltransferase in *Vitus vinifera*, improved cold tolerance in *A. thaliana* (Gao et al. 2019). Additionally, circRNAs potentially have roles in the biosynthesis of secondary metabolites. Researchers have explored the role of circRNAs in modulating SM biosynthesis in *S. miltiorrhiza*, using it as medicinal material in East Asian countries (Jiang et al. 2021). A total of 2,476 circRNAs from three types of plant tissues in *S. miltiorrhiza* were identified and analyzed. Brassinosteroid biosynthesis is mediated by the SMil_00026090 gene, which encodes 22alpha-hydroxylase. The gene SMIL_00014508 encodes ent-kaurenoic acid hydroxylase, which is involved in gibberellin biosynthesis. Moreover, *SmDXS2* displayed a significant correlation with its circRNA SMscf2473-46693-46978. A significant increase in the *SmDXS2* gene and its circRNA expression was observed in the roots compared to the leaves and stems, consistent with the accumulation of tanshinones in *S. miltiorrhiza*, suggesting that circRNAs might be involved in the biosynthesis of SMs. Considering the limited number of studies on medicinal plants circRNAs, the above-described functions might not reflect their general roles in the regulation of SM biosynthesis. Future functional studies will be useful for discovering their regulatory functions.

## Epigenetic regulation of secondary metabolism in medicinal plants

Epigenetics influences gene transcription and a variety of cellular processes, such as SMs production. Recent research in epigenetics has improved our understanding of epigenetic regulatory processes. The use of genetic manipulation to increase the production of SMs is insufficient, since epigenetic mechanisms regulate gene expression in ways that are still poorly understood (Sanchez-Muñoz et al. 2019). Epigenetics can be used to improve engineering strategies to control the expression of important genes, potentially increasing product yields. The broad definition of epigenetics implies that a wide variety of mechanisms are considered epigenetic. Epigenetic modifications such as DNA methylation have received a great deal of attention, and histone modifications are also critical for the regulation of gene expression (Kumar et al. 2017). These epigenetic modifications, including DNA methylation and histone modifications, affect gene expression in many eukaryotes, including plants (Bird 2007). Here, we will summarize the mechanisms underlying epigenetic modifications, focusing on how influences SM production.

## DNA methylation

DNA methylation occurs when a methyl group is added to the C5 position of a nucleotide (Kumar et al. 2017). Both prokaryotes and eukaryotes undergo this epigenetic modification (He et al. 2011). Many organisms such as plants can methylate only cytosine residues, but other nucleotides can be methylated under certain conditions (Heithoff et al. 1999). Two related mechanisms reduce the transcription rate when methylation occurs within a gene promoter. Firstly, the additional methyl groups prevent certain TFs from recognizing and binding to the DNA (Cedar and Bergman 2009). Additionally, DNA methylation attracts other factors that bind specifically to the methylated DNA and block TF binding (Vanyushin and Ashapkin 2011). In plants, methylation is further divided into two types: de novo methylation and maintenance methylation (Sanchez-Muñoz et al. 2019). Plants depend on DRM methyltransferases to silence transcriptional activity by de novo methylation (Pribylova et al. 2019). DNA methylation is heritable throughout multiple generations of organisms due to the robustness of the maintenance methylation pathway (Martienssen and Colot 2001).

Plant development and physiology are shaped and regulated by epigenetic modifications. Gene expression, transposon activity, and chromosome interactions are all known to be influenced by DNA methylation at cytosine positions (Zhang et al. 2018a). In plants, DNA methylation influences the development and response to environmental cues (Zhang et al. 2018a, b, c; Vidalis et al. 2016). DNA methylation patterns have been linked to gene expression both in cis and in trans, possibly through small RNAs in *A. thaliana* (Kooke 2019). Furthermore, DNA modifications are likely to influence gene expression and metabolite levels (Leung and Gaudin 2020).

Developmentally and environmentally regulated metabolic pathways in plants are thought to be further controlled by epigenetic factors. For example, the color of apple skin comes from anthocyanins whose biosynthesis is controlled by genes that are differentially methylated at cytosine bases (Li et al. 2019b). The production of specialized metabolites can be controlled in vitro by DNA cytosine methylation in non-model plants. For example, methylation profiles differed between wild and cultivated ginseng and were associated with differential accumulation of specialized metabolites (Hao and Xiao 2018). In addition, DNA methylation inhibitors are capable of increasing phenolic product biosynthesis in *S. miltiorrhiza* (Bunge) hairy root cultures (Yang et al. 2018). Furthermore, the methylation state was correlated with benzylisoquinoline indole alkaloids in different organs and cultivars of opium poppy (Bulut et al. 2020). To establish how DNA methylation regulates specialized pathways in medicinal plants, an integrative analysis of multi-omics data is still needed. Plants and animals exhibit varying levels of DNA methylation, which is a common biological phenomenon. Plants exhibit a variety of DNA methylation types, which may occur in any cytosine sequence context (H = A, C, or T) (He et al. 2011). Variations in DNA methylation levels between plant species are significant (Vidalis et al. 2016). CG methylation plays an epigenetic role in triterpenoid saponin biosynthesis, indicating that epigenetic changes in both of these gene families affect platycoside synthesis (Kim et al. 2020). A comprehensive analysis of DNA methylation revealed the role of DNA methylation in controlling specialized metabolism in *C. roseus* (Dugé de Bernonville 2020). It might be possible to improve plant secondary metabolite production for pharmaceutical applications by leveraging the potential coordination between epigenetics and hormonal control. Moreover, different DNA methylation marks were observed in the promoters of genes involved in secondary metabolism and photosynthesis between spontaneous and cultivar-dependent recovery (Pagliarani et al. 2020). Studies have shown that cultures maintained in suspension culture for a long period of time often contain more methylated genes in secondary metabolite pathways (Sanchez-Muñoz et al. 2019). Currently, this is a significant barrier to the large-scale production of plant secondary metabolites.

In *P. ginseng*, DNA methylation is involved in its domestication process and quality control. Functional analysis revealed DNA methylation is related to different genes, suggesting that DNA methylation contributes to domestication (Li et al. 2017). The accumulation of ginsenosides determines the quality of *P. quinquefolium*, and cold conditions play a vital role in this process. The DNA demethylation in tender leaves in early spring can be triggered by sufficient winter cold exposure, closely correlating with the accumulation of ginsenoside in the roots (Hao et al. 2020). In plants, cytosine-5 DNA methyltransferases are responsible for maintaining epigenetic modifications to cytosine DNA. Genome-wide analyses identified eight putative SmC5-MTases in *S. miltiorrhiza*. Additionally, transcript abundance analysis suggested that SmC5-MTases are functionally important for the stress response and secondary metabolism in *S. miltiorrhiza*. The findings of a previous study provided useful information to determine the role of DNA methylation in the development and SM biosynthesis in medicinal plants (Li et al. 2018). *Pinellia ternata* (Thunb.) Breit. (Araceae; Pinelliae Rhizoma) is a typical Chinese herbal medicine, and planting it in the shade can effectively increase its yield, and it is widely used in the Chinese market. A comparison was made in *P. ternata* grown under natural light and under shade, showing that shading induced 32.51% of the gene DNA methylation and 16.25% demethylation, indicating that variations in DNA methylation may contribute to the increased production of *P. ternata* under shading conditions (Shi et al. 2020). Another result showed that treatment of a *V*. *amurensis* cell culture with 5-azacytidine, which inhibits DNA methylation, increased resveratrol production two-fold (Kiselev et al. 2011).

## Histone modification

A covalent change in histone amino acids occurs when histones are acetylated, methylated, phosphorylated, and ubiquitinated. When histone acetylation occurs, DNA becomes more receptive to transcription factors that activate genes; conversely, histone deacetylation and partially methylated histone sites (e.g., K9 and K27) bind tightly to chromatin and inhibit gene expression (Liu et al. 2014; Strahl et al. 2000). Histone acetylation regulates the expression of genes, seed germination, morphogenesis, and stress response in plants (Liu et al. 2016). Epigenetic changes have been shown to affect a variety of plant growth and development processes, such as flowering, seed germination, and response to biotic and abiotic stress (Ahmad et al. 2010; Zhao et al. 2019). Since nonmodel plants have long growth cycles and abundant populations, progress in epigenetic studies of nonmodel plants is relatively slow. Research on epigenetic regulation of secondary metabolism has made significant progress over the last two decades due to the development of epigenetic research methods. This has improved our understanding of the epigenetic regulation of secondary metabolism in nonmodel plants. In recent years, epigenetic modifications have been found to be involved in secondary metabolism regulation in many plants. Anthocyanin accumulation in *Malus* leaves under conditions of Pi deficiency was co-modulated by miR399d and epigenetic modification (Peng et al. 2020).

We have made significant progress in uncovering anthocyanin biosynthesis and regulatory mechanisms, but our fundamental understanding of epigenetic regulation in this pathway is still unclear. JMJ25 from *Populus* has been identified as a gene involved in anthocyanin biosynthesis, and its role in anthocyanin biosynthesis has been characterized by genetic and biochemical approaches. MYB182 expression is negatively regulated by JMJ25 through methylation of chromatin and DNA, thereby repressing anthocyanin synthesis (Fan et al. 2018)

## Conclusions

The importance of medicinal plants SMs in various industries has aroused interest in regulating these metabolites through manipulation of their synthesis pathways. Natural compounds with high therapeutic properties can be produced using the numerous silent and cryptic pathways found in genomes of medicinal plants. There may be ways to manipulate these regulatory pathways to increase SM production. Furthermore, synthetic biology approaches such as the use of microbial or yeast heterologous hosts offer a promising platform for improving the biotechnological production of these compounds. Currently, microbes such as *E. coli*, *Saccharomyces cerevisiae*, and *Corynebacterium glutamicum*, which are widely used as chassis cells in microbial biotransformation, provide the opportunity to produce bioactive compounds that are more abundant than can be obtained from natural sources or chemical synthesis (Ajikumar et al. 2010; Paddon et al. 2013; Galanie et al. 2015; Luo et al. 2019). However, heterologous reconstruction is nearly impossible for extremely complex or unclear pathways, especially those containing multiple P450 reactions (McElroy and Jennewein 2018). Because of these obstacles to reconstructing complex pathways in heterologous microorganisms, plant systems are currently a much better route for synthesizing SMs (Zhu et al. 2021). However, whether in microorganisms or plants, the identification of biosynthetic pathways remains the biggest challenge for SM biosynthesis.

Many important SMs have been identified in different plant species over the past decades, and their biosynthesis pathways have been elucidated. Furthermore, it is extremely important to identify the genes involved in the biosynthesis and modification of SMs, especially those involved in their modification. Recent advances in the study of medicinal plants have been largely supported by improvements in high-throughput technology ‘omics’. From genomics, multiple clusters of SM biosynthetic genes were predicted, enhancing their genomic potential for the discovery of novel bioactive compounds. Additional “omics”, including transcriptomics, translatomics, interactomics, proteomics and metabolomics etc., are being utilized to establish a system-level understanding of the bioprocesses in medicinal plants, of which the epigenome layer is critical. In epigenomics, the goal is to learn how the environment influences the expression of genes, putting the other “omics layers” in a meaningful and pertinent biological context.

Currently, epigenetics has become an important tool for enhancing the concentration of bioactive compounds in medicinal plants. With the demand for novel drugs soaring, epigenetic modifiers have become more important as effective methods for identifying high-throughput natural products. A key function of epigenetic modifiers is to activate silent SM gene clusters that increase the production of bioactive compounds. The production of SMs in plant cell cultures is controlled by a variety of epigenetic mechanisms, including DNA methylation and histone modification, which can both result in decreased production of SMs in long-term plant cell culture. Although metabolic engineering approaches, including elicitation, overexpression of biosynthetic pathway genes, competing pathway knockouts, and transcription factor engineering, are effective tools for increasing secondary metabolite production, their effects are not stable as cultures age. This is a significant barrier in preventing reliable commercial production of more compounds using plant cell culture. Instead, epigenetic engineering combined with elicitation or TF engineering could greatly increase biosynthesis while reducing adverse effects such as decreased growth.

The production of SMs can be increased in the short-term using classical metabolic engineering techniques, but complementary epigenetic engineering techniques ensure that those changes remain stable over time and are not suppressed by compensatory regulatory systems. Additionally, due to the genomic complexity and lack of efficient transformation approaches for some medicinal plants, it is important to establish a powerful platform for metabolic engineering in hairy roots and suspension cells. Using higher throughput techniques for plant transformation, such as multiplex CRISPR/Cas9 approaches, creating cell lines with more complex genetic and epigenetic changes is now becoming a possibility. By taking this new approach, we will not only be able to offer more affordable plant cell culture products, but also bring new products to the market that are not currently available. By harnessing the power of epigenetic engineering, pharmaceuticals and other natural products produced by plant cell culture can become more affordable and accessible.

Some SMs accumulate in different tissues or organelles after biosynthesis, where they perform biological functions. These specialized metabolites are long-distance trafficked through transporter proteins, such as ATP binding cassette (ABC), multidrug and toxic compound extrusion (MATE), purine permease (PUP) families (Shitan et al. 2014). Current advances in genomics and multi-omics analysis have annotated some transporter genes in plant genomes. For example, three MATE proteins (CmMATE1, ClMATE1 and CsMATE1) have been identified that enhance the fitness of plants by secreting CuB, CuE and CuC (Zhong et al. 2022; Ma et al. 2023). Thus, in the future, the identification of metabolite transporters from medicinal plants will be cost-saving and time-saving of metabolic engineering, simplify the purification process, and allow compounds to be harvested by pumping them out of their cells.

### Supplementary Information

**Additional file 1: Table S1.** bHLH TFs involved in regulating secondary metabolism in plants.

**Additional file 2: Table S2.** MYB TFs involved in regulating secondary metabolism in plants.

**Additional file 3: Table S3.** WRKY TFs involved in regulating secondary metabolism in plants.

**Additional file 4: Table S4.** AP2/ERF TFs involved in regulating secondary metabolism in plants.

**Additional file 5: Table S5.** bZIPs involved in regulating secondary metabolism in plants.

## Data Availability

Not applicable.

## Abbreviations

ABA: Abscisic acid
ABC: ATP binding cassette
ABRE: ABA responsive element
ADS: Amorpha-4,11-diene synthase
ALDH: Aldehyde dehydrogenase
AP2/ERF: APETALA2/Ethylene response factor
bHLH: Basic helix-loop helix
BPF: Box P-binding factor
bZIP: Basic leucine zipper motif
C4H: Cinnamic acid 4-hydroxylase
C5-MTases: Cytosine-5 DNA methyltransferases
CBFs: CRT element binding factors
CCL: Cinnamate:CoA ligase
CHI: Chalcone isomerase
CHS: Chalcone synthase
circRNAs: Circular RNAs
CPT: Camptothecin
CRISPR: Clustered regularly interspaced short palindromic repeats
CuB: Cucurbitacin B
CuC: Cucurbitacin C
CuE: Cucurbitacin E
CYP: Cytochrome P450
DBR: Double bond reductase
DMAPP: Dimethylallyl pyrophosphate
DREB: Dehydration responsive element binding
FPS: Farnesyl pyrophosphate synthase
IPP: Isopentenyl pyrophosphate
F6H: Flavonoid 6-hydroxylase
GBF: G-box binding factor
GSW: Glandular trichome-specific WRKY
GPS: Geranylgeranyl pyrophosphate synthase
hc-siRNAs: Heterochromatic siRNAs
HPPD: 4-hydroxyphenylpyruvated dioxygenase
HSF: Heat shock transcription factor
HY5: Elongated hypocotyl 5
lncRNAs: Long non-coding RNAs
LMF: Light-mediated CPT biosynthesis factor
MATE: Multidrug and toxic compound extrusion
MeJA: Methyl jasmonate
MEP pathways: 2-C-methylerythritol 4-phosphate pathways
miRNAs: MicroRNAs
MYB: Myeloblastosis
MVA pathways: Mevalonic-acid pathways
NAC: NAM, ATAF, and CUC transcription factor
nat-siRNAs: Natural antisense-derived siRNAs
ncRNAs: non-coding RNAs
ONSEN: Copia-like retransposon
PAL: Phenylalanine ammonia lyase
PUP: Purine permease
RAS: RA synthase
SA: Salicylic acid
SE: Squalene epoxidase
siRNAs: Small interfering RNAs
SMs: Secondary metabolites
SS: Squalene synthase
SRPP: Small rubber particle protein
ta-siRNAs: Transacting siRNAs
TAT: Tyrosine aminotransferase
THCAS: Delta-9-tetrahydrocannabinolic acid synthase
TIA: Terpenoid indole alkaloid
TLR: TrichomeLess regulator
TFs: Transcription factors
UGT: Uridine diphosphate glycosyltransferase
WRKY: WRKYGQK motif-containing transcription factor
