# Supplementary material for: Multilayered regulation of secondary metabolism in medicinal plants
Source: Mol Hortic. 2023 Jun 6;3:11. doi: 10.1186/s43897-023-00059-y (PMC10514987; doi:10.1186/s43897-023-00059-y)
Supplement: Supplementary file 1 — Additional file 1: Table S1. bHLH TFs involved in regulating secondary metabolism in plants. [file 43897_2023_59_MOESM1_ESM.docx]

**Supplementary Table 1 bHLH TFs involved in regulating secondary metabolism in plants**

| Species | Name | Compound | Function | Reference |
| --- | --- | --- | --- | --- |
| *Artemisia annua* | AabHLH1 | Artemisinin | Activator | (Ji et al.,2014) |
| *Antirrhinum majus* | Delila | Red anthocyanin | Activator | (Goodrich et al.,1992) |
| *Betula platyphylla* | BpbHLH9 | Triterpene | Activator | (Yin et al.,2017) |
| *Coptis japonica* | CjbHLH1 | Quinoline alkaloids | Activator | (Yamada et al.,2011) |
| *Catharanthus roseus* | CrMYC1 | Strictosidine | Activator | (Chatel et al.,2003) |
| *Catharanthus roseus* | CrMYC2 | Terpenoid indole alkaloids | Activator | (Zhang et al.,2011) |
| *Catharanthus roseus* | BIS1 | Monoterpene indole alkaloid | Activator | (Van Moerkercke et al.,2015) |
| *Cucumis sativus* | B1 | Cucurbitacin c | Activator | (Shang et al.,2014) |
| *Cucumis sativus* | Bt | Cucurbitacin c | Activator | (Shang et al.,2014) |
| *Dahlia variabilis* | DvIVS | Anthocyanin | Activator | (Ohno et al.,2011) |
| *Eustoma Russellianum* | GtbHLH1 | Anthocyanin | Activator | (Nakatsuka et al.,2008) |
| *Erigeron breviscapus* | EbbHLH80 | Flavonoid | Activator | (Gao et al.,2022) |
| *Ginkgo biloba* | GbbHLHs | Flavonoid | Activator | (Zhou et al.,2020) |
| *Glycyrrhiza glabra* | bHLH3 | Triterpene saponins | Activator | (Tamura et al., 2018) |
| *Gerbera hybrida* | GhMYC1 | Anthocyanin | Activator | (Elomaa et al., 1998) |
| *Ipomoea purpurea* | IpIVS | Anthocyanin | Activator | (Park et al., 2007) |
| *Ipomoea tricolor* | ItIVS | Anthocyanin | Activator | (Park et al., 2012) |
| *Medicago truncatula* | TSAR1 | Nonhemolytic saponin | Activator | (Mertens et al., 2016) |
| *Medicago truncatula* | TSAR2 | Hemolytic saponin | Activator | (Mertens et al., 2016) |
| *Medicago truncatula* | TSAR3 | Hemolytic saponin | Activator | (Ribeiro et al.,2020) |
| *Phalaenopsis bellina* | PpbHLH4 | Monoterpene | Activator | (Chuang et al., 2018) |
| *Perilla frutescens* | MYC-RP/GP | Anthocyanin | Activator | (Gong et al., 1999) |
| *Petunia hybrida* | PhAN1 | Anthocyanin | Activator | (Quattrocchio et al., 1993) |
| *Petunia hybrida* | JAF13 | Anthocyanin | Activator | (Quattrocchio et al., 1998) |
| *Panax notoginseng* | PnbHLH1 | Triterpenoids | Activator | (Zhang et al., 2017) |
| *Quinoa* | TSARL1 | Triterpene | Activator | (Jarvis et al., 2017) |
| *Quinoa* | TSARL2 | Triterpene | Activator | (Jarvis et al., 2017) |
| *Salvia miltiorrhiza* | SmbHLH74 | Tanshinone | Activator | (Zhang et al., 2015) |
| *Salvia miltiorrhiza* | SmbHLH92 | Tanshinone | Activator | (Zhang et al., 2015) |
| *Salvia miltiorrhiza* | SmMYC2 | Phenolic acids | Activator | (Yang et al., 2017) |
| *Salvia miltiorrhiza* | SmbHLH37 | Salvianolic acid | Repressor | (Du et al., 2018) |
| *Taxus cuspidata* | TcJAMYC | Taxol | Activator | (Nims et al., 2015) |
| *Taxus cuspidata* | TcJAMYC1 | Taxol | Repressor | (Lenka et al., 2015) |
| *Taxus cuspidata* | TcJAMYC2 | Taxol | Repressor | (Lenka et al., 2015) |
| *Taxus cuspidata* | TcJAMYC4 | Taxol | Repressor | (Lenka et al., 2015) |
