# Supplementary material for: Multilayered regulation of secondary metabolism in medicinal plants
Source: Mol Hortic. 2023 Jun 6;3:11. doi: 10.1186/s43897-023-00059-y (PMC10514987; doi:10.1186/s43897-023-00059-y)
Supplement: Supplementary file 2 — Additional file 2: Table S2. MYB TFs involved in regulating secondary metabolism in plants. [file 43897_2023_59_MOESM2_ESM.docx]

**Supplementary Table 2. MYB TFs involved in regulating secondary metabolism in plants.**

| Species | Name | Compound | Function | Reference |
| --- | --- | --- | --- | --- |
| *Artemisia annua* | AaMYB1 | Artemisinin | Activator | (Matias-Hernandez et al. 2017) |
| *Artemisia annua* | AaMYB108 | Artemisinin | Activator | (Liu et al. 2022) |
| *Artemisia annua* | AaMYB15 | Artemisinin | Repressor | (Wu et al. 2021) |
| *Artemisia annua* | AaTLR1 | Artemisinin | Repressor | (Lv et al. 2022) |
| *Artemisia annua* | AaTLR2 | Artemisinin | Repressor | (Lv et al. 2022) |
| *Catharanthus roseus* | CrBPF1 | Terpenoid indole alkaloid | Repressor | (Li et al. 2015) |
| *Chrysanthemum morifolium* | CmMYB6 | Anthocyanin | Activator | (Liu et al. 2015b; Hong et al. 2019) |
| *Chrysanthemum morifolium* | CmMYB7 | Anthocyanin | Activator | (Hong et al. 2019) |
| *Chrysanthemum morifolium* | CmMYB9a | Anthocyanin | Activator | (Wang et al. 2022c) |
| *Chrysanthemum morifolium* | CmMYB21 | Anthocyanin | Repressor | (Wang et al. 2022b) |
| *Chrysanthemum morifolium* | CmMYB#7 | Anthocyanin | Repressor | (Xiang et al. 2019) |
| *Chrysanthemum morifolium* | CmMYB4 | Anthocyanin | Repressor | (Hong et al. 2019) |
| *Chrysanthemum morifolium* | CmMYB5 | Anthocyanin | Repressor | (Hong et al. 2019) |
| *Chrysanthemum morifolium* | CmMYB1 | Flavonoid | Repressor | (Zhu et al. 2013) |
| *Chrysanthemum morifolium* | CmMYB8 | Flavonoid | Repressor | (Zhu et al. 2020) |
| *Dendrobium officinale* | DoMYB26 | Volatile terpenoids | Activator | (Lv et al. 2022a) |
| *Dendrobium officinale* | DoMYB31 | Volatile terpenoids | Activator | (Lv et al. 2022a) |
| *Dendrobium officinale* | DoMYB29 | Volatile terpenoids | Repressor | (Lv et al. 2022a) |
| *Erigeron breviscapus* | EbMYBP1 | Flavonoid | Activator | (Zhao et al. 2022) |
| *Epimedium sagittatum* | EsAN2 | Anthocyanin | Activator | (Huang et al. 2016b) |
| *Epimedium sagittatum* | EsMYBA1 | Anthocyanin | Activator | (Huang et al. 2013) |
| *Epimedium sagittatum* | EsMYB9 | Flavonoid | Activator | (Huang et al. 2017) |
| *Epimedium sagittatum* | EsMYBF1 | Flavonol | Activator | (Huang et al. 2016a) |
| *Fagopyrum tataricum* | FtMYB18 | Anthocyanin | Repressor | (Dong et al. 2020) |
| *Fagopyrum tataricum* | FtMYB31 | Flavonoid | Activator | (Sun et al. 2019) |
| *Fagopyrum tataricum* | FtMYB45 | Flavonoid | Repressor | (Wen et al. 2022) |
| *Fagopyrum tataricum* | FtMYB6 | Flavonol | Activator | (Yao et al. 2020) |
| *Fagopyrum tataricum* | FtMYB1 | Proanthocyanidin | Activator | (Bai et al. 2014) |
| *Fagopyrum tataricum* | FtMYB2 | Proanthocyanidin | Activator | (Bai et al. 2014) |
| *Fagopyrum tataricum* | FtMYB18 | Proanthocyanidin | Repressor | (Dong et al. 2020) |
| *Fagopyrum tataricum* | FtMYB116 | Rutin | Activator | (Zhang et al. 2019) |
| *Fagopyrum tataricum* | FtMYB11 | Rutin | Repressor | (Zhou et al. 2017) |
| *Fagopyrum tataricum* | FtMYB13 | Rutin | Repressor | (Zhang et al. 2018a) |
| *Fagopyrum tataricum* | FtMYB14 | Rutin | Repressor | (Zhang et al. 2018a) |
| *Fagopyrum tataricum* | FtMYB15 | Rutin | Repressor | (Zhang et al. 2018a) |
| *Fagopyrum tataricum* | FtMYB16 | Rutin | Repressor | (Zhang et al. 2018a) |
| *Ginkgo biloba* | GbMYBFL | Flavonoid | Activator | (Zhang et al. 2018b) |
| *Ginkgo biloba* | GbMYBF2 | Flavonoid | Repressor | (Xu et al. 2014) |
| *Ginkgo biloba* | GbMYBR1 | Phenylpropanoid | Repressor | (Su et al. 2020) |
| *Glycyrrhiza uralensis* | GlMYB4 | Flavonoid | Activator | (Li et al. 2020) |
| *Glycyrrhiza uralensis* | GlMYB88 | Flavonoid | Activator | (Li et al. 2020) |
| *Gynostemma pentaphyllum* | GpMYB81 | Flavonol | Activator | (Huang et al. 2021) |
| *Gynostemma pentaphyllum* | GpMYB81 | Gypenoside | Activator | (Huang et al. 2021) |
| *Lonicera japonica* | LjaMYB12 | Flavonoid | Activator | (Qi et al. 2019) |
| *Lonicera macranthoides* | LmMYB15 | Chlorogenic acid | Activator | (Tang et al. 2021) |
| *Lonicera macranthoides* | LmMYB15 | Phenylpropanoid | Activator | (Tang et al. 2021) |
| *Lycium barbarum* | LbAN2 | Anthocyanin | Activator | (Zong et al. 2019) |
| *Lycium ruthenicum* | LrMYB1 | Flavonoid | Activator | (Wang et al. 2020) |
| *Lycium ruthenicum* | LrAN2 | Anthocyanin | Activator | (Zong et al. 2019; Ye et al. 2021) |
| *Ophiorrhiza pumila* | OpMYB1 | Camptothecin | Repressor | (Rohani et al. 2016) |
| *Panax ginseng* | PgMYB2 | Ginsenoside | Activator | (Liu et al. 2019b) |
| *Panax notoginseng* | PnMYB2 | Ginsenoside | Unclear | (Xia et al. 2022) |
| *Perilla frutescens* | PfMYB-P1 | Anthocyanin | Activator | (Gong et al. 1999) |
| *Pogostemon cablin* | PatSWC4 | Patchoulol | Activator | (Chen et al. 2020) |
| *Pueraria lobata* | PlMYB1 | Isoflavonoids | Activator | (Shen et al. 2021) |
| *Salvia miltiorrhiza* | SmMYB1 | Anthocyanin | Activator | (Zhou et al. 2021) |
| *Salvia miltiorrhiza* | SmMYB1 | Phenolic acid | Activator | (Zhou et al. 2021) |
| *Salvia miltiorrhiza* | SmPAP1 | Phenolic acid | Activator | (Hao et al. 2015) |
| *Salvia miltiorrhiza* | SmMYB98 | Phenolic acid | Activator | (Hao et al. 2020) |
| *Salvia miltiorrhiza* | SmMYB4 | Phenolic acid | Repressor | (Tian et al. 2022) |
| *Salvia miltiorrhiza* | SmMYB36 | Phenolic acid | Repressor | (Ding et al. 2017) |
| *Salvia miltiorrhiza* | SmMYB2 | Salvianolic acid | Activator | (Deng et al. 2020) |
| *Salvia miltiorrhiza* | SmMYB52 | Salvianolic acid B | Activator | (Yang et al. 2021) |
| *Salvia miltiorrhiza* | SmMYB111 | Salvianolic acid B | Activator | (Li et al. 2018) |
| *Salvia miltiorrhiza* | SmMYB9 | Tanshinone | Activator | (Zhang et al. 2017) |
| *Salvia miltiorrhiza* | SmMYB36 | Tanshinone | Activator | (Ding et al. 2017) |
| *Salvia miltiorrhiza* | SmMYB98 | Tanshinone | Activator | (Hao et al. 2020) |
| *Salvia miltiorrhiza* | SmMYB98b | Tanshinone | Activator | (Liu et al. 2019a) |
| *Salvia miltiorrhiza* | SmMYB4 | Tanshinone | Repressor | (Tian et al. 2022) |
| *Salvia miltiorrhiza* | SmMYB39 | Rosmarinic acid | Repressor | (Zhang et al. 2013) |
| *Scutellaria baicalensis* | SbMYB12 | Baicalin | Activator | (Wang et al. 2022a) |
| *Scutellaria baicalensis* | SbMYB8 | Flavonoid | Activator | (Yuan et al. 2014) |
| *Scutellaria baicalensis* | SbMYB2 | Phenylpropanoid | Activator | (Qi et al. 2015) |
| *Scutellaria baicalensis* | SbMYB7 | Phenylpropanoid | Activator | (Qi et al. 2015) |
| *Scutellaria baicalensis* | SbMYB12 | wogonoside | Activator | (Wang et al. 2022a) |
| *Taxus chinensis* | TcMYB29a | Paclitaxel | Activator | (Cao et al. 2022) |
| *Taxus media* | TmMYB3 | Paclitaxel | Activator | (Yu et al. 2020) |
| *Taxus media* | TmMYB39 | Paclitaxel | Activator | (Yu et al. 2022) |
