# Supplementary material for: Multilayered regulation of secondary metabolism in medicinal plants
Source: Mol Hortic. 2023 Jun 6;3:11. doi: 10.1186/s43897-023-00059-y (PMC10514987; doi:10.1186/s43897-023-00059-y)
Supplement: Supplementary file 3 — Additional file 3: Table S3. WRKY TFs involved in regulating secondary metabolism in plants. [file 43897_2023_59_MOESM3_ESM.docx]

**Supplementary Table 3 WRKY TFs involved in regulating secondary metabolism in plants.**

| Species | Name | Compound | Function | Reference |
| --- | --- | --- | --- | --- |
| *Artemisia annua* | AaWRKY1 | Artemisinin | Activator | (Chen et al.,2017) |
| *Artemisia annua* | AaWRKY9 | Artemisinin | Activator | （Fu et al.,2021） |
| *Artemisia annua* | AaWRKY4 | Artemisinin | Activator | (Huang.et al.,2021) |
| *Artemisia annua* | AaGSW2 | Artemisinin | Activator | (Xie et al.,2021) |
| *Artemisia annua* | AaGSW1 | Artemisinin, Dihydroartemisinic acid | Activator | (Xie et al.,2021) |
| *California poppy* | CjWRKY1 | Benzylisoquinoline alkaloid | Activator | (Yamada et al.,2021) |
| *Catharanthus roseus* | CrWRKY1 | Vinblastine | Activator | （Suttipanta et al.,2011) |
| *Cannabis sativa* | CsWRKY1 | Cannabinoids | Repressor | （Liu et al.,2021） |
| *Ophiorrhiza pumila* | OpWRKY6 | Pentacyclic quinoline alkaloid | Repressor | (Wang et al., 2022) |
| *Pogostemon cablin* | PcWRKY44 | Patchouli alcohol | Activator | （Wang et al.,2022) |
| *Panax ginseng* | PgWRKY4X | Ginsenosides | Activator | (Yao et al., 2020). |
| *Panax notoginseng* | PnWRKY9 | Notoginsenoside | Activator | (Zheng et al., 2022) |
| *Panax notoginseng* | PnWRKY22 | Salicylic acid | Activator | (Ning et al., 2021) |
| *Salvia miltiorrhiza* | SmWRKY61 | Tanshinone | Activator | (Chen et al.,2022) |
| *Salvia miltiorrhiza* | SmWRKY1 | Tanshinone | Activator | (Cao et al.,2018） |
| *Salvia miltiorrhiza* | SmWRKY2 | Tanshinone | Activator | (Deng et al.,2019) |
| *Salvia miltiorrhiza* | SmWRKY34 | Tanshinones,  Phenolic acids | Repressor | (Shi et al.,2022) |
| *Taxus chinensis* | TcWRKY8 | Taxol | Activator | (Zhang et al.,2018) |
| *Taxus chinensis* | TcWRKY47 | Taxol | Activator | (Zhang et al.,2018) |
