# Supplementary material for: Multilayered regulation of secondary metabolism in medicinal plants
Source: Mol Hortic. 2023 Jun 6;3:11. doi: 10.1186/s43897-023-00059-y (PMC10514987; doi:10.1186/s43897-023-00059-y)
Supplement: Supplementary file 4 — Additional file 4: Table S4. AP2/ERF TFs involved in regulating secondary metabolism in plants. [file 43897_2023_59_MOESM4_ESM.docx]

**Supplementary Table 4. AP2/ERF TFs involved in regulating secondary metabolism in plants.**

| **Species** | **Name** | **Compound** | **Function** | **Reference** |
| --- | --- | --- | --- | --- |
| *Catharanthus roseu* | CrERF5 | Anhydrovinblastine | Activator | (Pan et al. 2019) |
| *Catharanthus roseu* | CrERF5 | Ajmalicine | Activator | (Pan et al. 2019) |
| *Catharanthus roseu* | CrERF5 | Catharanthine | Activator | (Pan et al. 2019) |
| *Catharanthus roseu* | CR1 | Serpentine | Repressor | (Liu et al. 2017) |
| *Catharanthus roseu* | CrERF5 | Vinblastine | Activator | (Pan et al. 2019) |
| *Catharanthus roseu* | CrERF5 | Vindoline | Activator | (Pan et al. 2019) |
| *Catharanthus roseu* | CR1 | Vindoline | Repressor | (Liu et al. 2017) |
| *Panax notoginseng* | PnERF1 | *Ginsenoside (Rg3, Rh1, Rd, Rg1, F1 and Re)* | Activator | (Deng et al. 2016) |
| *Isatis indigotica* Fort. | Ii049 | Lignan | Activator | (Ma et al. 2017) |
| *Isatis indigotica* Fort. | (IiAP2/ERF063) | Lignan | Activator | (Xiao et al. 2023) |
| *Isatis indigotica* Fort. | Ii049 | Lignin | Activator | (Ma et al. 2017) |
| *Salvia miltiorrhiza* | SmERF128 | Cryptotanshinone | Activator | (Zhang et al. 2019) |
| *Salvia miltiorrhiza* | SmERF128 | Dihydrotanshinone I | Activator | (Zhang et al. 2019) |
| *Salvia miltiorrhiza* | SmERF128 | Tanshinone I | Activator | (Zhang et al. 2019) |
| *Salvia miltiorrhiza* | SmERF128 | Tanshinone IIA | Activator | (Zhang et al. 2019) |
