# Supplementary material for: Multilayered regulation of secondary metabolism in medicinal plants
Source: Mol Hortic. 2023 Jun 6;3:11. doi: 10.1186/s43897-023-00059-y (PMC10514987; doi:10.1186/s43897-023-00059-y)
Supplement: Supplementary file 5 — Additional file 5: Table S5. bZIPs involved in regulating secondary metabolism in plants. [file 43897_2023_59_MOESM5_ESM.docx]

**Supplementary Table 5. bZIPs involved in regulating secondary metabolism in plants.**

| **Species** | **Name** | **Compound** | **Function** | **Reference** |
| --- | --- | --- | --- | --- |
| *Artemisia annua* | AabZIP1 | Artemisinin | Activator | (Shu et al.,2022) |
| *Artemisia annua* | AabZIP9 | Artemisinin | Activator | (Shen et al.,2019) |
| *Artemisia annua* | AaABF3 | Artemisinin | Activator | (Zhong et al.,2018) |
| *Artemisia annua* | AaHY5 | Artemisinin | Activator | (Hao et al.,2019) |
| *Artemisia annua* | AaTGA6 | Artemisinin | Activator | (Lv et al.,2019) |
| *Artemisia annua* | AabZIP11 | Artemisinin | Activator | (Ma et al.,2019) |
| *Bupleurum chinense* | BcbZIP134 | Saikosaponin | Repressor | (Xu et al.,2019) |
| *Camptotheca acuminata* | CaLMF | Camptothecin | Repressor | (Chang et al.,2019) |
| *Catharanthus roseus* | CrGBF1 | Indolylalkylamine alkaloid | Repressor | (Sibéril et al.,2001) |
| *Catharanthus roseus* | CrGBF2 | Indolylalkylamine alkaloid | Repressor | (Sibéril et al.,2001) |
| *Fagopyrum tataricum* | FtHY5-1 | Flavonoid  Rutin, Quercetin, | Activator | (Wang et al.,2021) |
| *Fagopyrum tataricum* | FtHY5-2 | Flavonoid  Rutin, Quercetin | Activator | (Wang et al.,2021) |
| *Medicago truncatula* | MtbZIP17 | Triterpene saponins | Activator | (Ribeiro et al.,2022) |
| *Medicago truncatula* | MtbZIP60 | Triterpene saponins | Activator | (Ribeiro et al.,2022) |
| *Salvia miltiorrhiza* | SmbZIP1 | Phenolic acid | Activator | (Deng et al.,2020) |
| *Salvia miltiorrhiza* | SmbZIP2 | Phenolic acid | Repressor | (Shi et al.,2021) |
| *Salvia miltiorrhiza* | SmAREB1 | Phenolic acid,  Salvianolic acid | Activator | (Jia et al.,2017) |
| *Salvia miltiorrhiza* | SmbZIP1 | Tanshinone | Repressor | (Deng et al.,2020) |
| *Taraxacum brevicorniculatum* | TbbZIP1 | Polyterpenoids | Activator | (Fricke et al.,2013) |
| *Tripterygium wilfordii* | TwTGA1 | Serpentine, Catharanthine | Activator | (Han et al.,2020) |
| *Tripterygium wilfordii* | TwTGA1 | Vincaleukoblastine | Repressor | (Han et al.,2020) |
| *Vitis quinquangularis* | VqbZIP1 | Resveratrol | Activator | (Wang et al.,2019) |
